# Genus *Litophyton*: A Hidden Treasure Trove of Structurally Unique and Diversely Bioactive Secondary Metabolites

**DOI:** 10.3390/md21100523

**Published:** 2023-09-29

**Authors:** Xian-Yun Yan, Ling Zhang, Qi-Bin Yang, Zeng-Yue Ge, Lin-Fu Liang, Yue-Wei Guo

**Affiliations:** 1 College of Materials Science and Engineering, Central South University of Forestry and Technology, 498 South Shaoshan Road, Changsha 410004, China; yanxianyun@csuft.edu.cn (X.-Y.Y.); zling0710@csuft.edu.cn (L.Z.); yqb@csuft.edu.cn (Q.-B.Y.); gezengyue@csuft.edu.cn (Z.-Y.G.); 2 School of Medicine, Shanghai University, 99 Shangda Road, Bao Shan District, Shanghai 200444, China; 3 Shandong Laboratory of Yantai Drug Discovery, Bohai Rim Advanced Research Institute for Drug Discovery, 198 Binhai East Road, High-tech Zone, Yantai 264117, China; 4 Collaborative Innovation Center of Yangtze River Delta Region Green Pharmaceuticals, College of Pharmaceutical Science, Zhejiang University of Technology, Hangzhou 310014, China

**Keywords:** soft coral, *Litophyton*, secondary metabolites, terpenes, bioactivities, cytotoxicity

## Abstract

Marine soft corals are prolific sources of various natural products that have served as a wealthy reservoir of diverse chemical scaffolds with potential as new drug leads. The genus *Litophyton* contains almost 100 species but only a small proportion of them has been chemically investigated, which calls for more attentions from global researchers. In the current work, 175 secondary metabolites have been discussed, drawing from published data spanning almost five decades, up to July 2023. The studied species of the genus *Litophyton* resided in various tropical and temperate regions and encompassed a broad range of biologically active natural products including terpenes, steroids, nitrogen-containing metabolites, lipids, and other metabolites. A wide spectrum of pharmacological effects of these compounds had been evaluated, such as cytotoxic, antiviral, antibacterial, antifungal, anti-malarial, antifeedant, anti-inflammatory, molluscicidal, PTP1B inhibitory, insect growth inhibitory, and neuroprotective activities. This review aims to offer an up-to-date survey of the literature and provide a comprehensive understanding of the chemical structures, taxonomical distributions, and biological activities of the reported metabolites from the title genus whenever available.

## 1. Introduction

More than two-thirds of the Earth’s surface is covered by oceans, which harbor a vast array of creatures, including plants, animals, and microbes. Since the ancient times, marine organisms have been used as sources of foods [1], cosmetic ingredients [2], and drugs [3], which are hotspots for global researchers nowadays [4]. Continuous studies focused on the secondary metabolites derived from marine environments, resulting in a rapid expansion of marine natural products [5]. These substances displayed a wide spectrum of potential pharmacological effects, including antivirus [6], anti-osteoclastogenesis [7], antimicrobial [8], and antitumor [9]. To date, almost 20 drugs from marine sources are in clinical use [10].

The marine soft coral genus *Litophyton* belongs to the family Nephtheidae, order Alcyonacea, subclass Octocorallia. It might be worth pointing out the taxonomic relationship between the genera *Nephthea* and *Litophyton*, both of which are in the same family Nephtheidae. In 2016, the genus *Nephthea* was synonymized with the genus *Litophyton* due to their identical characteristics in terms of mitochondrial DNA molecular information and morphology (including features such as bone needle, tentacle shape, polyp, and stem) [11,12]. Currently, the genus *Litophyton* consists of nearly 100 species, according to the Word Register of Marine Species (WoRMS) [13]. They are widely distributed throughout tropical and temperate waters, such as the South China Sea [14], Red Sea [11], as well as other waters of the Indo-Pacific Ocean [15,16,17]. 

The alcyonarian *Litophyton viridis* was observed to provide chemical protection for the fish *Abudefduf leucogaster* [18]. In addition to the ecological role, the extracts of several soft corals of the genus *Litophyton* have been biologically screened and showed a variety of potent bioactivities, such as antioxidant [19], genotoxic [20], cytotoxic [19,21,22], HIV-1 enzyme inhibitory [21], antibacterial [22], anti-inflammatory [23], antifungal [24], and wound healing [25] activities. Chemical investigations on *Litophyton* soft corals were carried out by researchers worldwide and revealed that soft corals of the genus *Litophyton* are prolific producers of bioactive secondary metabolites. However, there was no specific review of compounds isolated from soft corals of the original *Litophyton* genus. However, a summary of the chemical constituents and biological properties of the synonymized *Nephthea* genus was reported [26,27], which covered the work published from 1974 to 2010. On the basis of an extensive literature search using SciFinder, this work specifically summarized for the first time all the secondary metabolites isolated from species currently classified within the genus *Litophyton*, covering a period of near five decades (between 1975 and July 2023) for the original *Litophyton* species and since 2011 for the synonymized *Nephthea* species.

## 2. Classification of Secondary Metabolites from the Genus *Litophyton*

Since the early reports of novel cembrane diterpenes from the soft corals *Nephthea* sp. [28] and *L. viridis* [29] in the beginning of 1970s, many research groups around the world have carried out chemical investigation of the genus *Litophyton*, resulting in fruitful achievements. For instance, two uncommon *bis*-sesquiterpenes, dikelsoenyl ether and linardosinene H, were encountered during the research of two alcyonarians, *Nephthea erecta* [30] and *Litophyton nigrum* [31], respectively. Up to July 2023, a total of 175 secondary metabolites have been isolated and characterized in *Litophyton* corals during almost 50 years of research (Table S1). These chemical compounds can be structurally classified as sesquiterpenes, sesquiterpene dimers, diterpenes, norditerpenes, tetraterpenes, meroterpenes, steroids, ceramides, pyrimidines, peptides, prostaglandins, *γ*-lactones, fatty acids, glycerol ethers, and selenides. In the following subsections, these compounds were further grouped under different categories based on their structural features. Among them, the ceramides, pyrimidines, and peptides were placed under one category, ‘nitrogen-containing metabolites’. The pack of ‘lipids’ comprise prostaglandins, *γ*-lactones, fatty acids, and glycerol ethers. Other metabolites include selenides. Herein, the chemical structures, taxonomical distributions, and biological activities of the reported metabolites from the title genus whenever available are described.

## 3. Sesquiterpenes

**Figure 1:**
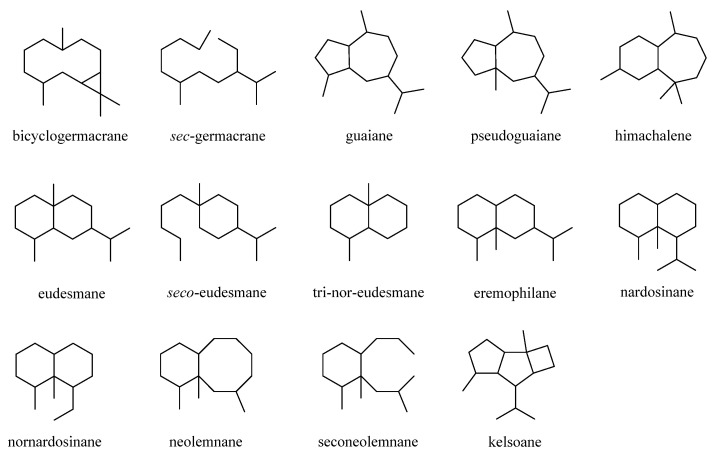
Carbon frameworks of the sesquiterpenes reported from soft corals of the genus *Litophyton*.

This was a large cluster of terpenes obtained from the genus *Litophyton* with an account of 38 compounds in this review. These compounds possessed a variety of carbon frameworks, which could be further classified into 14 categories: bicyclogermacrane, s*ec*-germacrane, guaiane, pseudoguaiane, himachalene, eudesmane, *seco*-eudesmane, tri-nor-eudesmane, eremophilane, nardosinane, nornardosinane, neolemnane, seconeolemnane, and kelsoane (Figure 1). This diversity of skeletons makes sesquiterpenes the most interesting type of natural products from this genus. The different sesquiterpenes were distributed in four species, *Litophyton arboreum*, *L. nigrum*, *Litophyton setoensis*, *Nephthea erecta*, and an unclearly identified *Nephthea* sp., which inhabited different marine environments including the Red Sea, South China Sea, and the waters around Indonesia and Taiwan (Table S1).

### 3.1. Bicyclogermacrane Sesquiterpenes

**Figure 2:**
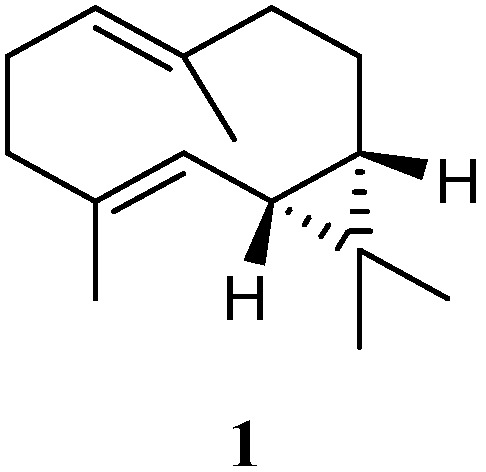
Chemical structure of the bicyclogermacrane sesquiterpene isolated from soft corals of the genus *Litophyton*.

Chemical investigation of the soft coral *L. arboreum*, which was collected near Bali, Indonesia, yielded the sesquiterpene (−)-bicyclogermacrene (**1**) [32] (Figure 2). This compound exhibited low antiproliferative activities against the cell lines L-929 and K-562 with GI_50_ values of 186 and 200 μM, respectively, and low cytotoxic effect against the HeLa cell line with CC_50_ of 182 μM.

### 3.2. Sec-Germacrane Sesquiterpenes

**Figure 3:**
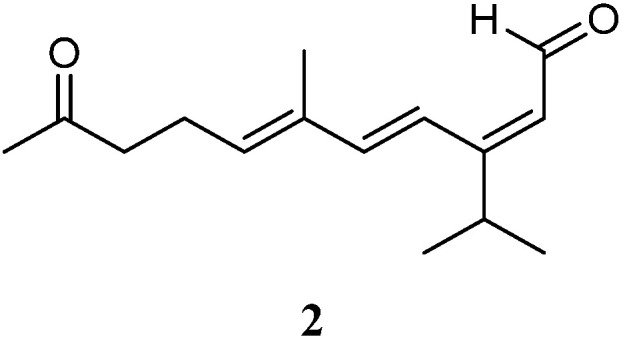
Chemical structure of the s*ec*-germacrane sesquiterpene from soft corals of the genus *Litophyton*.

Very recently, Ahmed et al. [33] carried out chemical investigation of the Red Sea specimen *L. arboreum*, which was collected at Neweba, Egypt. The acyclic sesquiterpene (2*E*,6*E*)-3-isopropyl-6-methyl-10-oxoundeca-2,6-dienal (**2**) was found from this sample, which possessed a *sec*-germacrane nucleus (Figure 3). Anti-malarial bioassays disclosed the isolate **2** was active against chloroquine-sensitive (D6) and chloroquine-resistant (W2) strains of *Plasmodium falciparum* with IC_50_ values of 3.7 and 2.2 mg/mL, respectively. In addition, the metabolite **2** was non-toxic to the Vero cell line at the concentration of 4.76 mg/mL. These findings demonstrated that sesquiterpene **2** could be developed as an anti-malarial lead compound that is highly safe in the range of tested concentrations.

### 3.3. Guaiane Sesquiterpenes

Interestingly, the guaiane sesquiterpenes were frequently encountered in the Red Sea soft coral *L*. *arboreum*.

**Figure 4:**
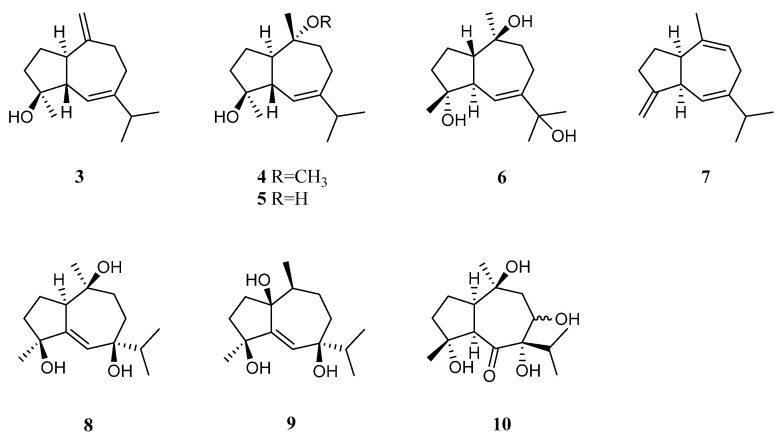
Chemical structures of the guaiane sesquiterpenes from soft corals of the genus *Litophyton*.

Bioassay-guided fractionation of the Red Sea alcyonarian *L. arboreum* by Ellithey et al., which was collected at Sharm El-Sheikh, Egypt, yielded three guaiane sesquiterpenes alismol (**3**), 10-*O*-methyl alismoxide (**4**), and alismoxide (**5**) [34] (Figure 4). Compound **3** showed potent inhibitory activity against HIV-1 protease receptor with IC_50_ of 7.2 µM, compared to the positive control, which had IC_50_ of 8.5 μM. A molecular docking study disclosed the hydrogen bond between **3** and the amino acid residue Asp 25 in the hydrophobic receptor pocket with a score of −11.14. Meanwhile, sesquiterpenes **3** and **4** showed moderate cytotoxic activities against the cell lines HeLa (IC_50_ 30 and 38 μM, respectively) and Vero (IC_50_ 49 and 49.8 μM, respectively). Moreover, **4** exhibited moderate cytotoxicity against the U937 cell line with IC_50_ of 50 µM. However, **5** was judged as inactive against the above-mentioned cell lines (all IC_50_ > 100 µM). In a further study, compounds **2** and **5** demonstrated cytostatic action in HeLa cells, revealing potential use in virostatic cocktails. In Ellithey’s continual study [35], alismol (**3**) showed promising cytotoxic effects against the cancer cell lines HepG2, MDA and A549 (IC_50_ 4.52, 7.02, and 9.23 μg/mL, respectively).

Hawas’s group reported the presence of alismol (**3**) in a Red Sea specimen of *L. arboreum* collected off the coast of Jeddah, Saudi Arabia, together with another guaiane sesquiterpene alismorientol B (**6**) [36] (Figure 4). These two secondary metabolites were subjected to antimicrobial and cytotoxic bioassays. As a result, metabolites **3** and **6** showed weak to strong antibacterial activities against *Escherichia coli* ATCC 10536, *Pseudomonas aeruginosa* NTCC 6750, *Bacillus cereus* ATCC 9634, *Bacillus subtilis* ATCC6633, and *Staphylococcus aureus* ATCC5141 with MIC values ranging from 10.4 to 1.3 μg/mL. Here, compound **6** had significant activity against *B. cereus* ATCC 9634 with MIC of 1.3 μg/mL. Compounds **3** and **6** exhibited weak to moderate antifungal activities against *Candida albicans* and *Aspergillus niger* with MIC values ranging from 10.1 to 6.0 μg/mL. Moreover, they displayed cytotoxic effects against the cell lines MCF-7, HCT-116, and HepG2, with IC_50_ ranging from 4.32 to 44.52 μM. Here, compound **6** showed the most potent cytotoxic effect against MCF-7 cells with IC_50_ of 4.32 μM. Additionally, Hawas’s group evaluated the methanolic extract of the above-mentioned soft coral for its in vivo genotoxicity and antigenotoxicity against the mutagenicity induced by the anticancer drug cyclophosphamide [20]. The extract was found to be safe and nongenotoxic at 100 mg/kg b. wt. Moreover, the mice group of cyclophosphamide pretreated with the extract (100 mg/kg b. wt.) showed significant reduction in the percentage of chromosomal aberrations induced in bone marrow and mouse spermatocytes.

The existence of alismoxide (**5**) was shown in the Egyptian Red Sea *L. arboreum* collection from Hurghada by Mahmoud et al. [37]. In the anticancer bioassays, sesquiterpene **5** displayed no cytotoxic activities against the cell lines A549, MCF-7, and HepG2 (all IC_50_ > 100 µmol/mL). The co-existence of alismol (**3**) and alismoxide (**5**) as well as an undescribed sesquiterpene, litoarbolide A (**7**), and three known analogues 4*α*,7*β*,10*α*-trihydroxyguai-5-ene (**8**), leptocladol B (**9**), and nephthetetraol (**10**) (Figure 4) in another Egyptian Red Sea *L. arboreum* specimen from Neweba, was revealed by Ahmed et al.’s work [33]. Viewing from the perspective of their structures, litoarbolide A (**7**) was supposed to be the biosynthetic precursor to other sesquiterpenes, which could be generated via further post-translational modifications. The anti-malarial properties of substances **7**–**10** were evaluated. However, only compounds **9** and **10** exhibited anti-malarial activities against chloroquine-resistant *P. falciparum* W2 with IC_50_ values of 4.3 and 3.2 mg/mL, respectively.

Guaiane sesquiterpenes 10-*O*-methyl alismoxide (**4**) and alismoxide (**5**) were also obtained from the octocoral *Nephthea* sp. by Hegazy et al., which was collected from the Egyptian Red Sea off the coast of Hurghada [38]. These two metabolites showed cytotoxicity against the cell line MCF-7 (IC_50_ 85.5 and 151.9 μg/mL, respectively).

### 3.4. Pseudoguaiane Sesquiterpenes

**Figure 5:**
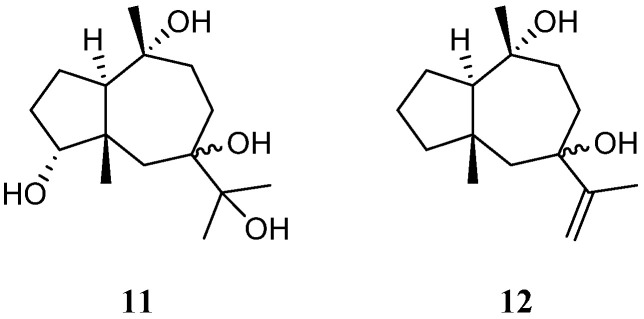
Chemical structures of the pseudoguaiane sesquiterpenes from soft corals of the genus *Litophyton*.

A new pseudoguaiane-type sesquiterpene named litopharbol (**11**) (Figure 5) was isolated from the methanolic extract of the Saudi Arabian Red Sea soft coral *L. arboreum* by Hawas’s group [36]. Its structure was determined through the elucidation of NMR data. Compound **11** exhibited a wide spectrum of antibacterial activities against Gram-negative bacteria *E. coli* ATCC 10536 and *P. aeruginosa* NTCC 6750, as well as Gram-positive bacteria *B. cereus* ATCC 9634, *B. subtilis* ATCC6633, and *S. aureus* ATCC5141 with MIC values ranging from 1.8 to 9.6 μg/mL. Among these bacteria, **11** showed significant activity against *B. cereus* ATCC 9634 with an MIC of 1.8 μg/mL. In addition, this sesquiterpene exhibited weak antifungal activities against *C. albicans* and *A. niger* with MIC values of 12.5 and 12.9 μg/mL, respectively. Moreover, it displayed cytotoxic effects against cell lines MCF-7, HCT-116, and HepG2 with IC_50_ values of 9.42, 26.21, and 38.92 μM, respectively. In Hawas’s continual study, litopharbdiol (**12**) was identified, which shared the same carbon framework with **11** [20] (Figure 5). However, no bioassay for this compound was reported in the article.

### 3.5. Himachalene Sesquiterpenes

**Figure 6:**
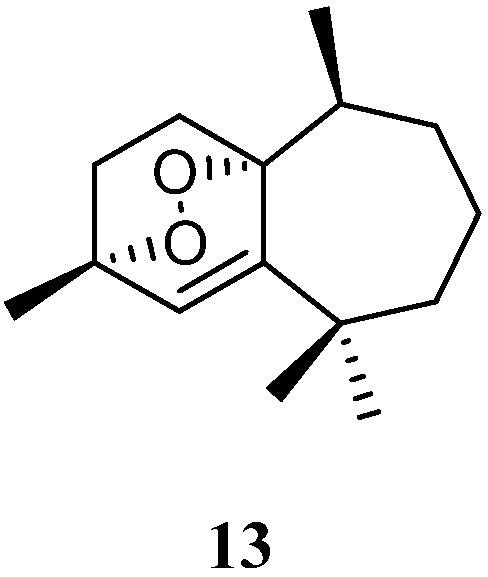
Chemical structure of the himachalene sesquiterpene from soft corals of the genus *Litophyton*.

Purification of the CH_2_Cl_2_/MeOH extract of Saudi Arabian Red Sea alcyonarian *L. arboreum* yielded a new himachalene-type sesquiterpene 3*α*,6*α*-epidioxyhimachal-1-ene (**13**) (Figure 6), which showed antiproliferative effects toward three different cancer cell lines MCF-7, HCT116, and HepG-2 [39]. (It might be worth pointing out that no specific data of the bioassay results were provided in this article).

### 3.6. Eudesmane Sesquiterpenes

**Figure 7:**
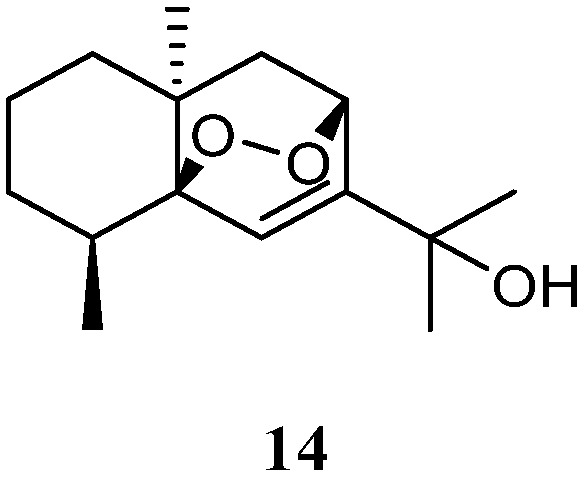
Chemical structure of the eudesmane sesquiterpene from soft corals of the genus *Litophyton*.

The *n*-hexane-chloroform (1:1) fraction of the Egyptian Red Sea *L. arboreum* sample exhibited cytotoxicity towards the A549 cell line (IC_50_ 22.6 mg/mL) [37]. The subsequent bioassay-guided isolation yielded a eudesmane sesquiterpene 5*β*,8*β*-epidioxy-11-hydroxy-6-eudesmene (**14**) (Figure 7). Compound **14** exerted noticeable activity against the A549 cell line (IC_50_ 67.3 µmol/mL) compared to etoposide as standard cytotoxic agent (IC_50_ 48.3 µmol/mL). However, this compound did not show cytotoxic effects against cell lines MCF-7 and HepG2 (both IC_50_ > 100 µmol/mL).

### 3.7. Seco-Eudesmane Sesquiterpenes

**Figure 8:**
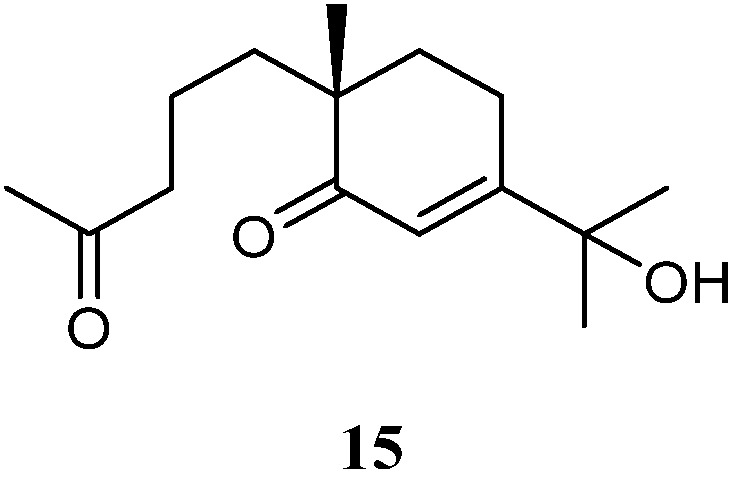
Chemical structure of the *seco*-eudesmane sesquiterpene from soft corals of the genus *Litophyton*.

In the above-mentioned study [37], a *seco*-eudesmane sesquiterpene chabrolidione B (**15**) (Figure 8) was co-isolated. However, compound **15** was judged as inactive against the cell lines A549, MCF-7, and HepG2 (all IC_50_ > 100 µmol/mL).

### 3.8. Tri-Nor-Eudesmane Sesquiterpenes

**Figure 9:**
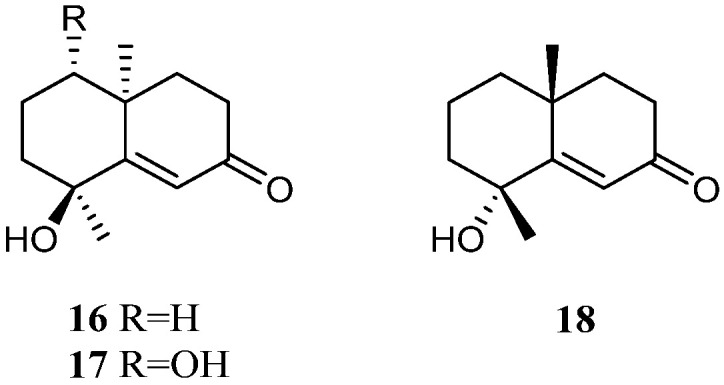
Chemical structures of the tri-nor-eudesmane sesquiterpenes from soft corals of the genus *Litophyton*.

The methanolic extract of the Saudi Arabia Red Sea *L. arboreum* collection harbored two tri-nor-eudesmane sesquiterpenes teuhetenone A (**16**) and calamusin I (**17**) [36] (Figure 9). Interestingly, these two nor-sesquiterpenes **16** and **17** displayed a wide spectrum of bioactivities. In the antibacterial bioassays, they showed moderate to strong activities against *E. coli* ATCC 10536, *P. aeruginosa* NTCC 6750, *B. cereus* ATCC 9634, *B. subtilis* ATCC6633, and *S. aureus* ATCC5141 with MIC values ranging from 10.9 to 1.2 μg/mL. Here, **16** exhibited the most potent activity against *E. coli* ATCC 10536 with an MIC of 1.9 μg/mL, and **17** displayed the most potent activity against *P. aeruginosa* NTCC 6750 with an MIC of 1.2 μg/mL. In the antifungal biotests, they exhibited weak to moderate activities against *C. albicans* and *A. niger* with MIC values ranging from 7.4 to 3.2 μg/mL. In the cytotoxic experiments, they displayed cytotoxic effects against cell lines MCF-7 and HepG2 with IC_50_ ranging from 6.43 to 39.23 μM. In addition, the methanolic extract of the Egyptian Red Sea *L. arboreum* sample yielded another tri-nor-eudesmane sesquiterpene 7-oxo-tri-nor-eudesm-5-en-4*β*-ol (**18**) [37] (Figure 9). However, this nor-sesquiterpene **18** did not show cytotoxic activities against the cell lines A549, MCF-7, and HepG2 (all IC_50_ > 100 µmol/mL).

### 3.9. Eremophilane Sesquiterpenes

**Figure 10:**
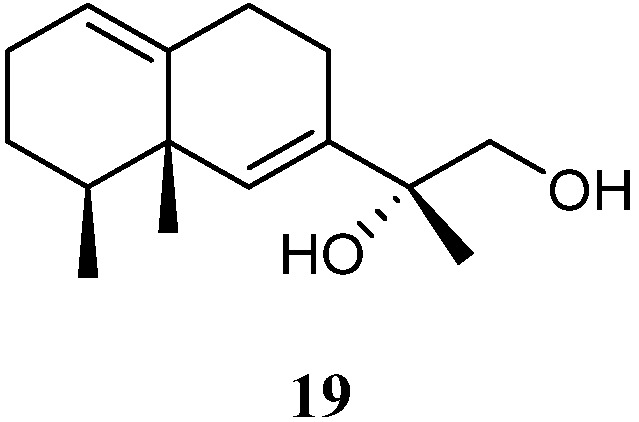
Chemical structure of the eremophilane sesquiterpene from soft corals of the genus *Litophyton*.

11,12-Dihydroxy-6,10-eremophiladiene (**19**) (Figure 10) was obtained from the soft coral *L. nigrum*, using a structure-oriented HR-MS/MS approach [31]. This alcyonarian specimen was collected at Xisha Islands, Hainan, China. However, no bioassays were performed due to its scarcity.

### 3.10. Nardosinane Sesquiterpenes

Interestingly, the South China Sea soft coral *L. nigrum* is a rich source of nardosinane sesquiterpenes.

**Figure 11:**
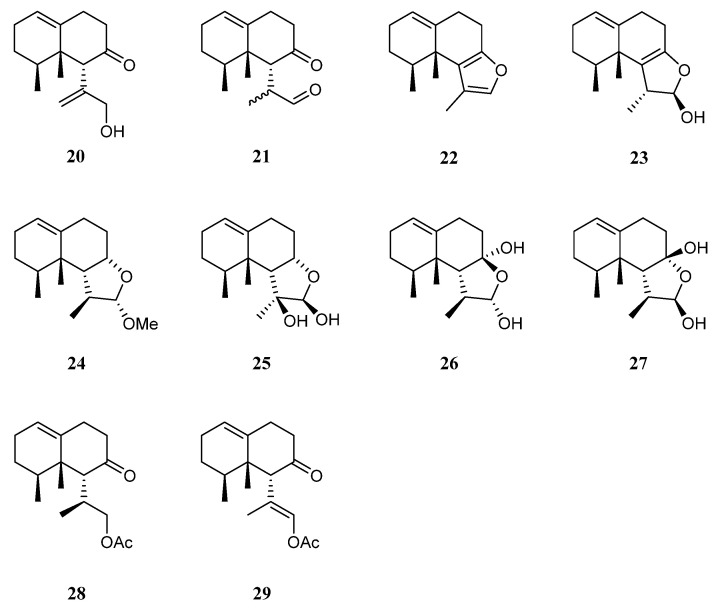
Chemical structures of the nardosinane sesquiterpenes from soft corals of the genus *Litophyton*.

The chemical investigation of the Xisha collection by Yang et al. afforded two new terpenes linardosinenes B (**20**) and C (**21**) [14] (Figure 11). These two compounds were evaluated for cytotoxities against different cell lines. Sesquiterpene **20** exhibited cytotoxic effect against the THP-1 cell line with IC_50_ of 59.49 μM, while compound **21** displayed cytotoxicities against the cell lines SNU-398 and HT-29 with IC_50_ of 24.3 and 44.7 μM, respectively. In their continual study on the Xisha sample, four additional new secondary metabolites linardosinenes D–G (**22**–**25**) (Figure 11) were obtained [40]. All metabolites exhibited weak inhibitory effect against bromodomain-containing protein 4 (BRD4), a promising therapeutic target in various human diseases, at a concentration of 10 μM with inhibitory rates ranging from 15.8% to 18.1%.

Using a structure-oriented HR-MS/MS approach, an undescribed sesquiterpene linardosinene I (**26**), along with its known 7*β*,12*α*-epimer lemnal-l(l0)-ene-7*β*,12*α*-diol (**27**) (Figure 11) were isolated from Xisha alcyonarian *L. nigrum* [31]. The absolute configuration of terpene **27** was determined to be 4*S*,5*S*,6*R*,7*S*,11*S*,12*S* by single crystal X-ray diffraction analysis with Cu K*α* radiation [Flack parameter: 0.13(14)]. Sesquiterpene **26** exhibited a potent PTP1B inhibitory activity (IC_50_ 10.67 μg/mL). It also showed moderate cytotoxic activities against the human tumor cell lines HT-29, Capan-1, and SNU-398 with IC_50_ values of 35.48, 42.55, and 25.17 μM, respectively. However, co-isolated metabolite **27** was inactive against PTP1B (IC_50_ > 20 μg/mL) or cell lines HT-29, Capan-1, and SNU-398 (all IC_50_ > 50 μM).

Recently, two members of this cluster, paralemnolin J (**28**) and (l*S*,8*S*,8a*S*)-*l*-[(*E*)-2′-acetoxy-l′-methylethenyl]-8,8*a*-dimethyl-3,4,6,7,8,8*a*-hexahydronaphthalen-2(1*H*)-one (**29**) (Figure 11), were isolated in the chemical investigation of a Balinese soft coral *L. setoensis* [16]. In terms of biological activity, cytotoxic effects against several solid tumor and leukemia cell lines HT-29, Capan-1, A549, and SNU-398 were assessed for compounds **28** and **29**. As a result, both compounds showed weak cytotoxic activities against the test cell lines (all IC_50_ > 20 μM).

### 3.11. Nornardosinane Sesquiterpenes

**Figure 12:**
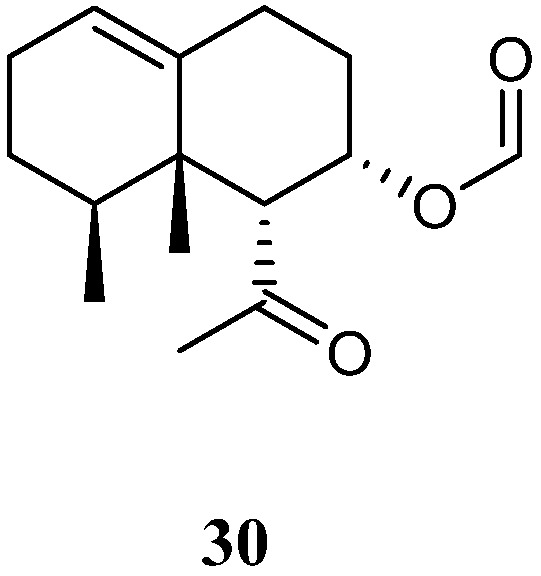
Chemical structure of the nornardosinane sesquiterpene from soft corals of the genus *Litophyton*.

Chemical study of Xisha alcyonarian *L. nigrum* afforded an uncommon nornardosinane sesquiterpene linardosinene A (**30**) [14] (Figure 12). The absolute configuration of **30** was determined by a modified Mosher’s method and TDDFT ECD approach. This isolate was evaluated for cytotoxicity against the THP-1 cell line and inhibitory activities against the PTP1B, BRD4, HDAC1, and HDAC6 protein kinases. However, it was inactive against the above-mentioned cell line and protein kinases.

### 3.12. Neolemnane Sesquiterpenes

**Figure 13:**
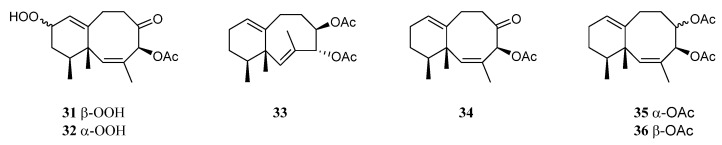
Chemical structures of the neolemnane sesquiterpenes from soft corals of the genus *Litophyton*.

A study on the chemical constituents of the Chinese soft coral *L. nigrum* yielded three new sesquiterpenes lineolemnenes A–C (**31**–**33**), which possessed the neolemnane carbon framework, together with the related known compound 4-acetoxy-2,8-neolemnadien-5-one (**34**) [14] (Figure 13). It might be worth pointing out that the absolute configuration of **34** was unambiguously determined to be 1*S*,4*S*,12*S* by X-ray diffraction analysis for the first time. The cytotoxicities of substances **31** and **32** against SNU-398, HT-29, Capan-1, and A549 were evaluated. This revealed that **31** and **32** only exhibited cytotoxic activity against SNU-398 with IC_50_ values of 44.4 and 27.6 μM, respectively, and none of them showed potent inhibitory activities against the PTP1B, BRD4, HDAC1, and HDAC6 protein kinases. Compound **34** was also found in the Indonesian soft coral *L. setoensis*, together with another sesquiterpene paralemnolin E (**35**) [16] (Figure 13). They were subjected to cytotoxic bioassays against several solid tumor and leukemia cell lines HT-29, Capan-1, A549, and SNU-398. The results revealed both two compounds had weak cytotoxic activities against the test cell lines (all IC_50_ > 20 μM). Parathyrsoidin E (**36**) (Figure 13) was reported in the soft coral *Nephthea* sp., which was collected from the Egyptian coasts of the Red Sea at Sharm El-Sheikh [41]. In silica study indicated this compound was a potential SARS-CoV-2 main protease inhibitor.

### 3.13. Seconeolemnane Sesquiterpenes

**Figure 14:**
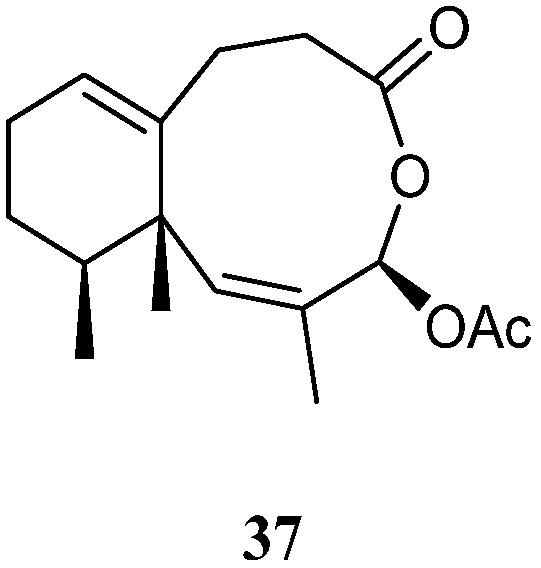
Chemical structure of the seconeolemnane sesquiterpene from soft corals of the genus *Litophyton*.

A new sesquiterpene lineolemnene D (**37**) (Figure 14) was isolated and characterized from the Xisha soft coral *L. nigrum* [14]. Structurally, this compound possessed an unusual seconeolemnane skeleton. The absolute configuration of **37** was determined to be 1*S*,4*R*,12*S* by TDDFT ECD approach. Bioassays including cytotoxicity against the THP-1 cell line and inhibitory activities against the PTP1B, BRD4, HDAC1, and HDAC6 protein kinases were performed for this isolate. However, it was judged as inactive in these biotests.

### 3.14. Kelsoane Sesquiterpenes

**Figure 15:**
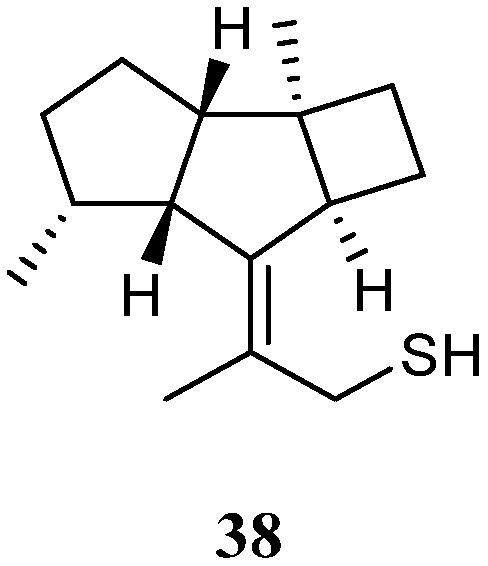
Chemical structure of the kelsoane sesquiterpene from soft corals of the genus *Litophyton*.

Interestingly, a new kelsoane-type sesquiterpene, namely kelsoenethiol (**38**) (Figure 15), was obtained from the Formosan soft coral *N. erecta* [30]. Its structure was elucidated with the assistance of quantum chemical calculations. The cytotoxicities of **38** against A-459, P-388, and HT-29 cancer cell lines were evaluated in vitro. The results revealed compound **38** exhibited cytotoxic activities against P-388 and HT-29 cells with ED_50_s of 1.3 and 1.8 μg/mL, respectively.

## 4. *Bis*-Sesquiterpenes

**Figure 16:**
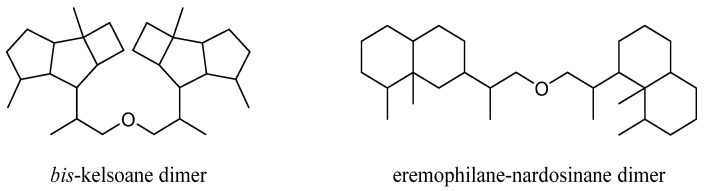
Carbon frameworks of the *bis*-sesquiterpenes from soft corals of the genus *Litophyton*.

This group of terpenes were extremely uncommon secondary metabolites identified from the genus *Litophyton* with only two members (Table S1). They were described as two subgroups according to their respective monomers: *bis*-kelsoane dimer and eremophilane-nardosinane dimer (Figure 16). All of them were the most unique type of natural products from this genus, since they were only obtained from the octocorals *N. erecta* and *L. nigrum* (Table S1).

### 4.1. Bis-Kelsoane Dimers

**Figure 17:**
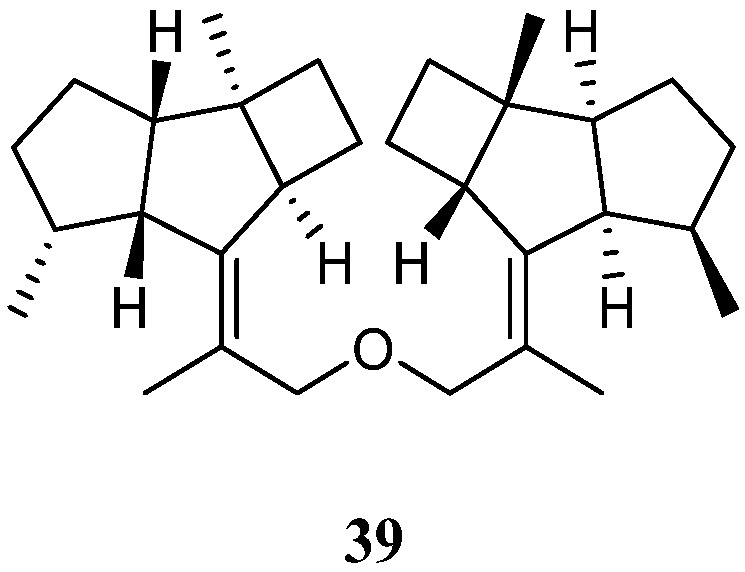
Chemical structure of the kelsoane-type *bis*-sesquiterpene from soft corals of the genus *Litophyton*.

Interestingly, a new kelsoane-type *bis*-sesquiterpene, namely dikelsoenyl ether (**39**) (Figure 17), was obtained from the Formosan soft coral *N. erecta* [30]. Its structure was elucidated with the assistance of quantum chemical calculations. The cytotoxicties of **38** against A-459, P-388, and HT-29 cancer cell lines were evaluated in vitro. However, it was judged as inactive.

### 4.2. Eremophilane-Nardosinane Bis-Sesquiterpenes

**Figure 18:**
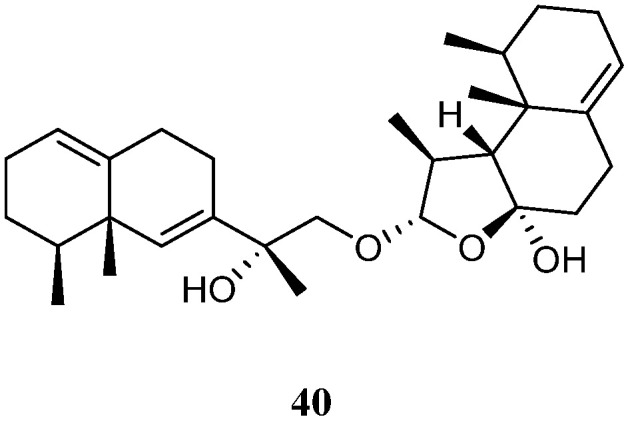
Chemical structure of the eremophilane-nardosinane *bis*-sesquiterpene from soft corals of the genus *Litophyton*.

Interestingly, one uncommon sesquiterpe dimer, linardosinene H (**40**) (Figure 18), was found in the soft coral *L. nigrum* collected at Xisha Islands, South China Sea, whose structure consisted of an eremophilane sesquiterpene **19** and a nardosinane sesquiterpene **26** [31]. Contrast to its monomer **26**, this bis-sesquiterpene **40** did not exhibit inhibitory activity against PTP1B (IC_50_ > 20 μg/mL) or the cell lines HT-29, Capan-1, A549, and SNU-398 (all IC_50_ > 20 μM).

## 5. Diterpenes

**Figure 19:**
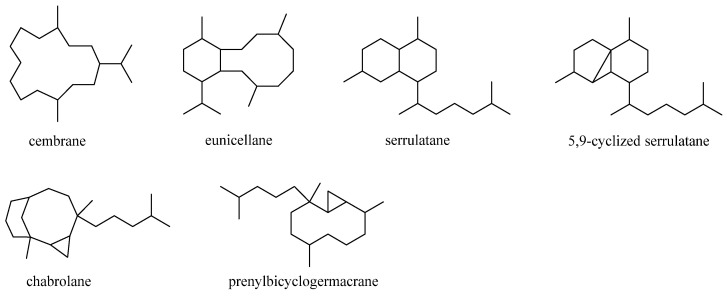
Carbon frameworks of the diterpenes reported from soft corals of the genus *Litophyton*.

Diterpenes were the largest cluster of terpenes consisting of 46 compounds. Structurally, this category of secondary metabolites could be divided into six subgroups: cembranes, eunicellanes, serrulatanes, 5,9-cyclized serrulatanes, chabrolanes, and prenylbicyclogermacranes (Figure 19). Analysis of taxonomical distributions revealed they were obtained from *L. viridis*, *L. arboreum*, *Litophyton viscudium*, *L. setoensis*, *Nephthea columnaris*, *Nephthea chablrolii*, and unclearly indentified *Litophyton* sp. and *Nephthea* sp., which were collected in the Red Sea and the waters around Indonesia, Taiwan, Malaysia, and Japan (Table S1).

### 5.1. Cembrane Diterpenes

**Figure 20:**
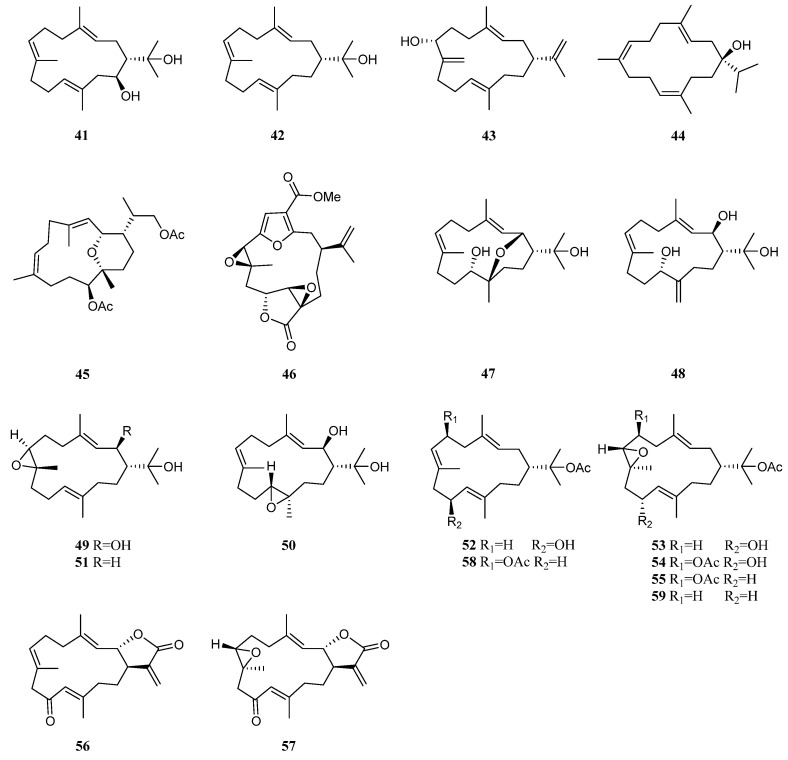
Chemical structures of the cembrane diterpenes from soft corals of the genus *Litophyton*.

In 1975, Tursch et al. reported the isolation and structure elucidation of a new compound 2-hydroxynephtenol (**41**) and its known analogue nephtenol (**42**) (Figure 20) from the alcyonarian *L. viridis*, collected off Serwaru (Leti Island, Maluku Province, Indonesia) [29]. Based on the chemical transformation, the absolute configuration of **42** was determined as 1*R*. These two terpenoids were also obtained from the Bornean octocoral *Nephthea* sp. [42]. Biological evaluation revealed they did not exhibit repellent activity against the maize weevil *Sitophilus zeamais* at 250 μg/cm^2^.

A new cembrane diterpene (3*E*,11*E*)-cembra-3,8(19),11,15-tetraene-7*α*-ol (**43**) (Figure 20), along with the known nephthenol (**42**), was isolated from the Red Sea soft coral *L. arboreum*, which was collected from Hurghada, Egypt [43]. The relative configuration of **43** was determined as 1*R*,7*R*. The (3*E*)- and (11*E*)-configurations were determined by comparison of the ^13^C chemical shifts for C-18 and C-20 methyl signals (<20.0 ppm). The biogenetical pathway of new terpene **43** from structurally related metabolite **42** was proposed in this work. Intrestingly, nephthenol (**42**) was also found in another Red Sea sample of *L. arboreum* collected from Jeddah coast, Saudi Arabia [20].

Chemical investigation of the chemical constituents of another Egyptian specimen *L. arboreum* collected from Sharm El-Sheikh led to the discovery of sarcophytol M (**44**) [34] (Figure 20). Compound **44** displayed a wide spectrum of bioactivities. It showed weak inhibitory activity against HIV-1 protease receptor with IC_50_ of 15.7 µM, compared to the positive control, which had IC_50_ of 8.5 μM. A molecular docking study disclosed the hydrogen bond between **44** and the amino acid residue Asp 25 in the hydrophobic receptor pocket with a score of −14.44, and sesquiterpene **44** showed moderate cytotoxic activities against the cell lines HeLa (IC_50_ 27.5 μM), Vero (IC_50_ 22 μM), and U937 (IC_50_ 31.7 μM).

Sarcophytol M (**44**) co-existed with a pyrane-based cembranoid 11-acetoxy-15,17-dihydroxy-2,12-epoxy-(3*E*,7*E*)-1-cembra-3,7-diene (**45**) (Figure 20) in the extract of Saudi Arabian alcyonarian *L. arboreum* [39]. Both compounds displayed antiproliferative effects toward cancer cell lines MCF-7, HCT116, and HepG-2 in comparison with standard anticancer drug (Doxorubicin). Here, **45** showed significant antiproliferative activities against the cell lines MCF-7, HCT116, and HepG2 (IC_50_ 19.1, 22.0, 24.0 μM, respectively). Further investigation on the possible mechanism of action had been conducted. The results showed **45** significantly increased the G_0_/G_1_ non-proliferating cell fraction from 55.42% to 68.98% with a compensatory decrease in cell populations in S-phase and G_2_/M-phase from 31.99% to 21.99% and from 10.82% to 7.63%, respectively.

Chemical study of the soft coral *L*. *arboreum*, collected near Bali, Indonesia, afforded a furanocembranoid diterpene 11*β*,12*β*-epoxypukalide (**46**) (Figure 20) [32]. This diterpene **46** showed low antiproliferative activities against the cell lines L-929 and K-562 (both GI_50_ > 129 μM), and low cytotoxic effect against the HeLa cell line (CC_50_ 115 μM).

Chemical investigation of the octocoral *N. columnaris*, collected off the waters of Taiwan, yielded four new 15-hydroxycembranoid diterpenes, namely columnariols A (**47**) and B (**48**) [44], 2*β*-hydroxy-7*β*,8*α*-epoxynephthenol (**49**), and 2*β*-hydroxy-11*α*,12*β*-epoxynephthenol (**50**), along with a new natural cembrane, epoxynephthenol (**51**) [45] (Figure 20). In the anti-inflammatory effects test, cembranes **47** and **48** were found to significantly inhibit the accumulation of the pro-inflammatory iNOS and COX-2 protein of the lipopolysaccharide (LPS)-stimulated RAW264.7 macrophage cells [44]. The cytotoxicities of compounds **47**–**51** against the proliferation of a panel of tumor cell lines, including MOLT-4, SUP-T1, U-937, DLD-1, LNCaP, and MCF7 were also studied [44,45]. However, only **47** exhibited moderate cytotoxicity toward LNCaP cells with an IC_50_ value of 9.80 μg/mL [44].

Three new cembranoid diterpenes, 10-hydroxy-nephthenol acetate (**52**), 7,8-epoxy-10-hydroxy-nephthenol acetate (**53**), and 6-acetoxy-7,8-epoxy-10-hydroxy-nephthenol acetate (**54**), along with a known compound, 6-acetoxy-7,8-epoxy-nephthenol acetate (**55**), were isolated from the Bornean soft coral *Nephthea* sp. [46] (Figure 20). These four isolates were subjected to antibacterial activity test against four antibiotic resistant bacterial strains *S*. *aureus* ATCC 6538, *Listeria monocytogenes* ATCC 19113, *E*. *coli* ATCC 35210, and *Salmonella typhimurium* ATCC 13311, and three cancer cell lines HeLa, MCF-7, and HT-29. As a result, compound **52** exhibited potent antibacterial activity against *S. aureus* and *E. coli* with an MBC/MIC ratio of 2.4 and 3.0, respectively, indicating a bactericidal antibiosis. On the other hand, compound **53** exhibited bacteriostatic antibiosis with a ratio of 6.0 against both the bacteria. Suppression of Hela and MCF-7 cell lines by compounds **52** and **53** was observed with IC_50_ values ranging from 25.0 to 125.0 μg/mL.

Further study on the Bornean alcyonarian *Nephthea* sp. led to the discovery of three new cembrane diterpenes, nephthecrassocolides A and B (**56** and **57**) and 6-acetoxy nephthenol acetate (**58**), along with three known compounds, nephthenol (**42**), 6-acetoxy-7,8-epoxy nephthenol acetate (**55**), and epoxy nephthenol acetate (**59**) [47] (Figure 20). All isolated compounds **41** and **54**–**58** displayed different levels of antifungal activities against *Exophiala* sp. NJM 1551, *Fusarium moniliforme* NJM 8995, *Fusarium oxysporum* NJM 0179, *Fusarium solani* NJM 8996, *Haliphthoros sabahensis* IPMB 1402, *Haliphthoros milfordensis* IPMB 1603, and *Lagenidium thermophilum* IPMB 1401. The most active compounds were **41** and **55** with an MIC value of 12.5 μg/mL against hyphal inhibition of *L. thermophilum* IPMB 1401.

### 5.2. Eunicellane Diterpenes

**Figure 21:**
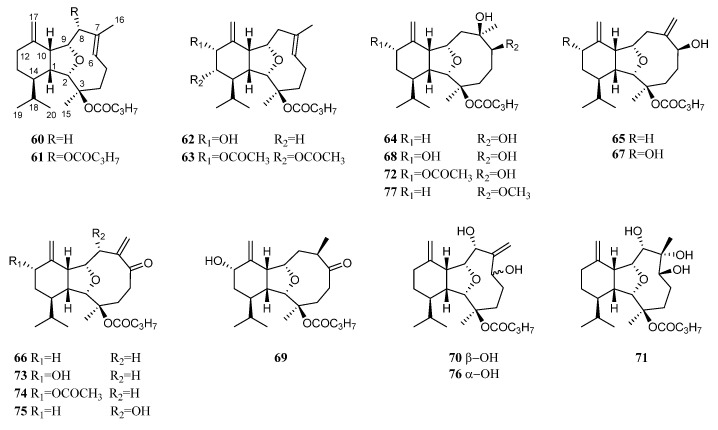
Chemical structures of the eunicellane diterpenes from soft corals of the genus *Litophyton*.

In 1987, Ochi et al. reported eunicellane diterpenes from the *Litophyton* animals for the first time. They were litophynins A (**60**) and B (**61**) (Figure 21) from the soft coral *Litophyton* sp., which was collected from a shallow area of Sukumo Bay in Kochi Prefecture, Japan [48]. Their structures were fully characterized by extensive 2D NMR studies and molecular mechanics calculations. Structurally, **81** was the butyric ester derivative of **60**. In the artificial diet feeding bioassay, they exhibited insect growth inhibitory against the silkworm, *Bombyx mori* L., with ED_50_ values of 12 and 2.7 ppm, respectively.

Inspired by this work, Ochi et al. performed further investigations on the insect growth inhibitory diterpenoids from the previously studied alcyonarian *Litophyton* sp., leading to the discovery of an array of new eunicellane diterpenes including litophynins C (**62**) [49], D (**63**) [50], E (**64**) [50], F (**65**) [51], G (**66**) [51], H (**67**) [51], I (**68**) [52], and J (**69**) [52] (Figure 21). The differences among their structures were mainly at the segment C-6, C-7 and C-16, which usually formed a double bond Δ^6^ (*endo*), or Δ^7(16)^ (*exo*) accompanied with a hydroxy or a ketone at C-6. The hydroxylation or acetylation at C-12/C-13 was also observed. The absolute configuration of litophynin C (**62**) was determined by analysis of CD spectrum of its *p*-bromobenzoate, based on the exciton chirality method of allylic alcohol benzoate [49]. Similarly, the absolute configuration of litophynin D (**63**) was determined by an application of the dibenzoate chirality rule [50].

Interestingly, these diterpenes exhibited various bioactivities. Litophynins C (**62**) and G (**66**) displayed insect growth inhibitory activity against the second instar larvae of the silkworm *B. mori* L. (ED_50_ 25 [49] and 42 [51] ppm, respectively). Litophynin D (**63**) exhibited significant brine shrimp lethality (LD_50_ 0.9 ppm) [50]. Litophynins I (**68**) and J (**69**) possess significant molluscicidal and repellent activities against the muricid gastropod *Drupella fragum* [52]. At 30 ppm concentration, diterpenes **68** and **69** exhibited 100% mortality to the snail within 24 h. They were also repellent to the gastropod when impregnated on filterpaper by 45 μg/cm^2^. These compounds, in combination with a wide variety of compounds stored in skin glands of *Litophyton* sp., appeared to be the foundation of a chemical defense adaptation to survive in predator-rich environments.

Litophynin C (**62**) (Figure 21) was also reported in the soft coral *Nephthea* sp., which was collected from the Egyptian coasts of the Red Sea at Sharm El-Sheikh [41]. In silica study indicated this compound was a potential SARS-CoV-2 main protease inhibitor.

Miyamoto et al. investigated the chemical constituents of the mucus secreted by the soft coral *Litopbyton* sp., which was collected from the rocky coast of Nango-cho, Miyazaki Prefecture, Japan [53]. In this study, two new eunicellin-type diterpenoids, litophynols A (**70**) and B (**71**), and three known diterpenoids litophynins E (**64**), H (**67**), and I monoacetate (**72**) (Figure 21) were identified. The absolute configurations of litophynols A (**70**) and B (**71**) were determined by application of the CD exciton chirality method, while the absolute configuration of litophynin E (**64**) was assigned by the Mosher’s method. Additionally, the absolute configurations of litophynin E (**64**) and litophynol B (**71**) were further confirmed by the application of the octant rule to their ozonolysis products, respectively. Interestingly, it was found that these five eunicellin-based diterpenoids were also present in the animal bodies of *Litopbyton* sp. but in low yields compared with the mucus. The performed bioassays revealed these five isolates were positive in a hemolytic reaction test, and crude diterpenoid fractions exhibited ichthyotoxicity (IC_100_ 20 ppm). This suggests that this soft coral holds eunicellin-type diterpenoids in its mucus for the purpose of defense against predators.

Iwagawa et al. found that the CH_2_Cl_2_-soluble portion of the MeOH extract of the Japanese alcyonarian *L*. *viscudium* showed moderate cytotoxic activity (IC_50_ = 6.9 μg/mL) against the proliferation of human promyelocytic leukemia cells (HL-60) [17]. Study on the chemical compositions of this species yielded five new eunicellin-type diterpenes, 6-oxo litophynin H (**73**), 6-oxo litophynin H 12-acetate (**74**), 6-oxo litophynol A (**75**), 6-*epi* litophynol A (**76**), and 6-methyl litophynol E (**77**), together with a previously reported litophynin F (**65**) (Figure 21) [17]. These secondary metabolites exhibited different levels of cytotoxicities against HL-60. Diterpenes **73** and **74** having a hydroxyl group or acetoxyl group at C-12 showed moderate cytotoxic activities (both IC_50_ 20 μM), while compound **75** possessing an additional hydroxyl group at C-8 and its reduced derivative **76** exhibited significant cytotoxic activities (IC_50_ 5.7 and 4.2 μM, respectively). The C-6 methoxyl and C-7 hydroxyl groups dramatically reduced the toxicity of diterpene **77** (IC_50_ 50 μM). Compound **75** with the absence of a hydroxyl group at C-8 and the presence of a *β*-hydroxyl group at C-6 displayed much less cytotoxic activity (IC_50_ 18 μM) than that of **76**.

### 5.3. Serrulatane Diterpenes

**Figure 22:**
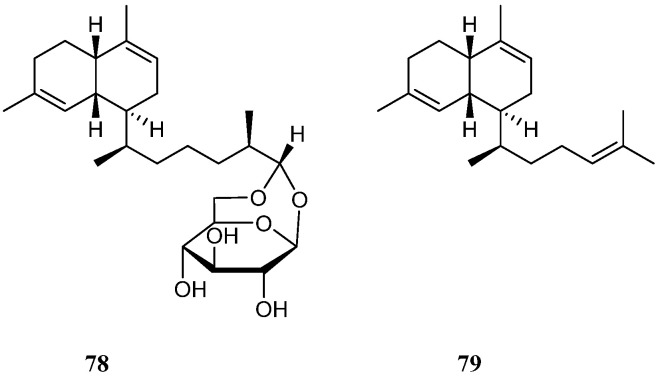
Chemical structures of the serrulatane diterpenes from soft corals of the genus *Litophyton*.

Two secondary metabolites lemnabourside (**78**) and biflora-4,9,15-triene (**79**) (Figure 22), which possessed the serrulatane carbon framework, were obtained from the soft coral *L. setoensis* collected along the coast of Singaraja, Bali Island, Indonesia [16]. In the bioassays, compounds **78** and **79** showed weak cytotoxic activities against the test cell lines HT-29, Capan-1, A549, and SNU-398 (all IC_50_ > 20 μM).

### 5.4. 5,9-Cyclized Serrulatane Diterpenes

**Figure 23:**
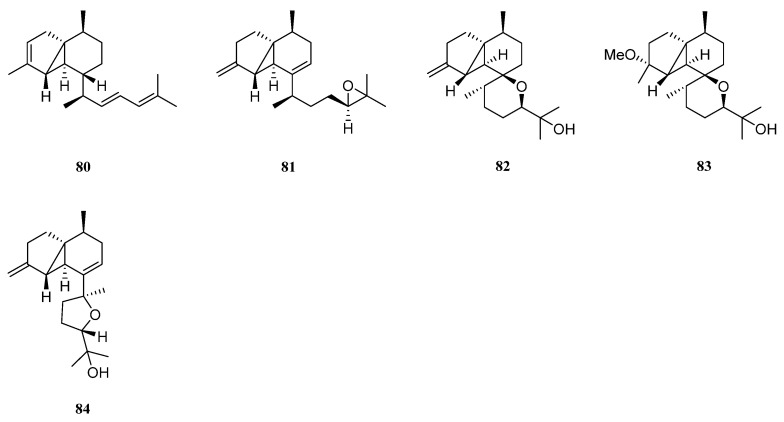
Chemical structures of the 5,9-cyclized serrulatane diterpenes from soft corals of the genus *Litophyton*.

Interestingly, five new diterpenes, litosetoenins A–E (**80**–**84**) (Figure 23), were isolated from a Balinese alcyonarian *L. setoensis* [16]. Their structures were elucidated by extensive spectroscopic analysis, quantum mechanical nuclear magnetic resonance approach, and chemical transformations. All of them possessed a rearranged serrulatane-type backbone with an unusual tricyclo[3.0.4]decane core. Moreover, **82**–**84** displayed intriguing tetracyclic backbones bearing either an additional tetrahydropyran or tetrahydrofuran ring, which were unprecedented and unique. All the isolates were subjected to the cytotoxic bioassays against cell lines HT-29, Capan-1, A549, and SNU-398. As a result, all the metabolites showed weak cytotoxic activities against these cell lines with IC_50_ values > 20 μM.

### 5.5. Chabrolane Diterpenes

**Figure 24:**
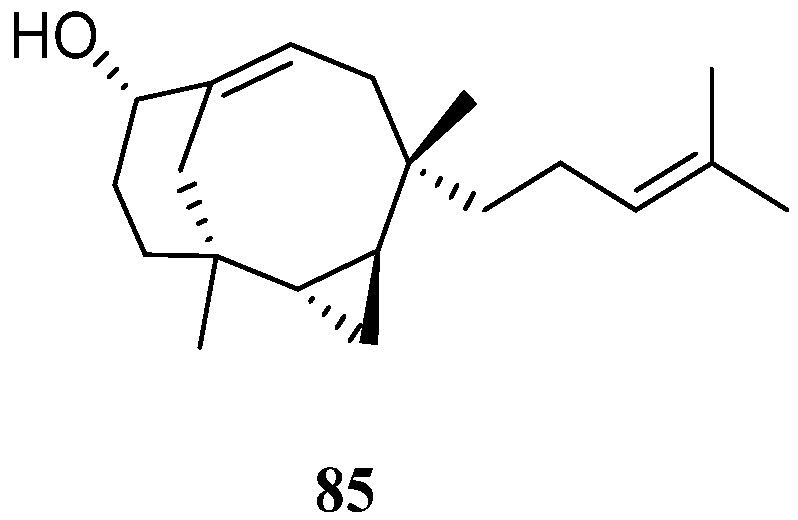
Chemical structure of the chabrolane diterpene from soft corals of the genus *Litophyton*.

Cytotoxicity-guided fractionation of the ethyl acetate extract of the soft coral *N*. *chablrolii* led to the isolation of a novel diterpene, chabrolin A (**85**) [54] (Figure 24). This secondary metabolite possessed an unprecedented terpenoid skeleton, which was tentatively named chabrolane. Compound **85** displayed cytotoxicity against P-388, with ED_50_ value of 3.18 μg/mL. However, **85** was not cytotoxic to A549 and HT-29 cell lines. Diterpene **85** was also examined for the antiviral activity against HCMV, but it was inactive.

### 5.6. Prenylbicyclogermacrane Diterpenes

**Figure 25:**
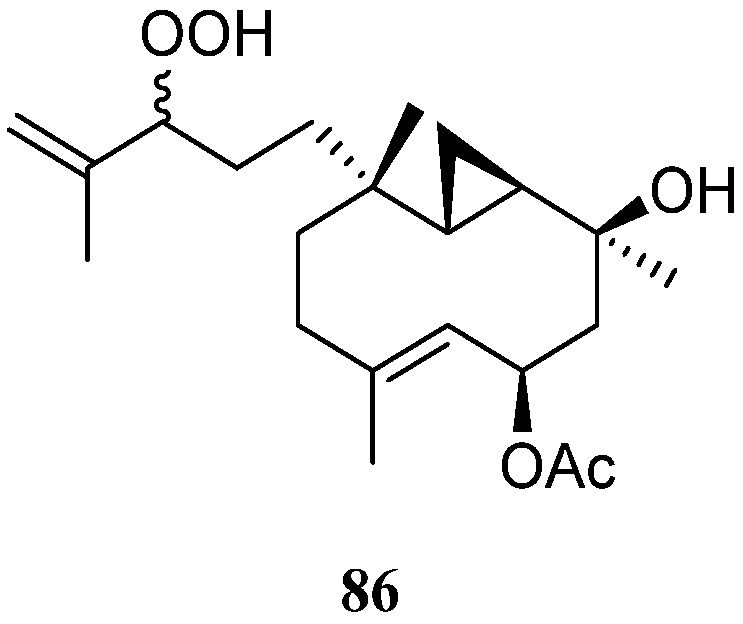
Chemical structure of the prenylbicyclogermacrane diterpene from soft corals of the genus *Litophyton*.

The prenylbicyclogermacrane-type diterpene, pacificin H (**86**) (Figure 25), was found in the soft coral *Nephthea* sp., which were collected from the Egyptian coasts of the Red Sea at Sharm El-Sheikh [41]. In silica study indicated this compound was a potential SARS-CoV-2 main protease inhibitor.

## 6. Norditerpenes

**Figure 26:**
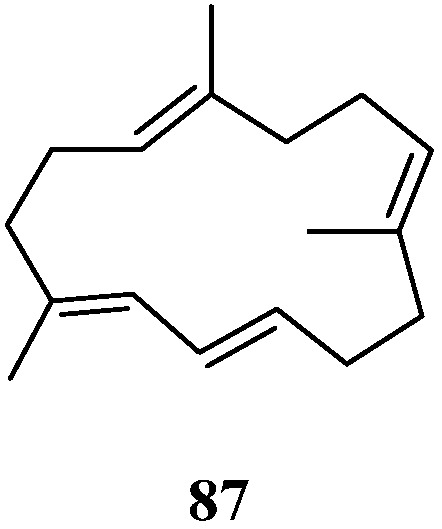
Chemical structure of the norditerpene from soft corals of the genus *Litophyton*.

A new norditerpene, chabrolene (**87**) (Figure 26), was isolated from *Nephthea* sp. collected from Mantanani Island, Sabah, Malaysia [42]. It might be worth pointing out that natural C_17_ compound with a 14-membered cyclic tetraene is extremely rare. This was the second report of a C_17_ norditerpene with a 14-membered ring from marine organisms. Compound **87** exhibited repellent activity against the maize weevil *S. zeamais* at 25 μg/cm^2^.

## 7. Tetraterpenes

**Figure 27:**
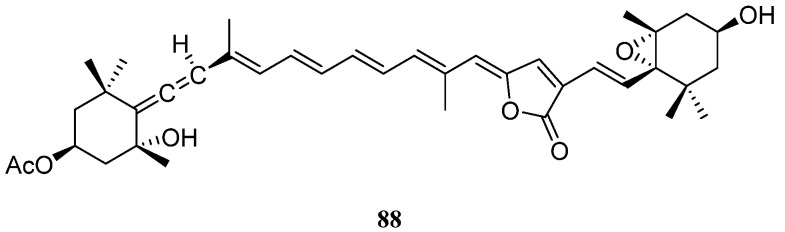
Chemical structure of the tetraterpene from soft corals of the genus *Litophyton*.

As revealed in literature, there was only one member of tetraterpene found in the genus *Litophyton*. That was all-*trans*-peridinin (**88**) (Figure 27), obtained from the Red Sea soft coral *L. arboreum* [43]. Terpene **88** showed moderate antiproliferative activities against cell lines HUVEC and K-562 (GI_50_ 48.4 and 53.8 μM, respectively), and moderate cytotoxicity against the HeLa cell line (IC_50_ 51.9 μM).

## 8. Meroterpenes

**Figure 28:**
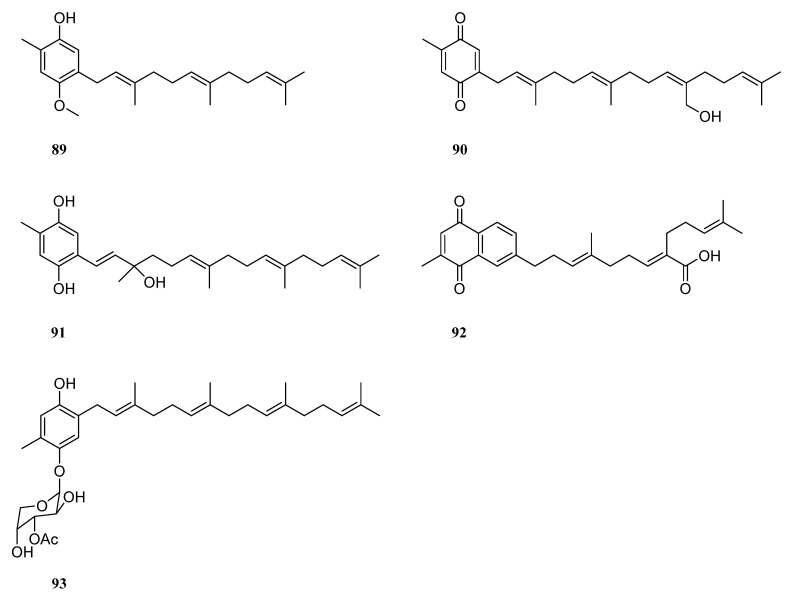
Chemical structures of the meroterpenes from soft corals of the genus *Litophyton*.

Four meroterpenes, *O*-methylisogrifolin (**89**), chabrolobenzoquinone E (**90**), chabrolohydroxybenzoquinone E (**91**), chabrolonaphthoquinone A (**92**), and nephthoside monoacetate (**93**), were identified from the Red Sea soft coral *Nephthea* sp. [41] (Figure 28). In silica studies indicated that these compounds were potential SARS-CoV-2 main protease inhibitors.

## 9. Steroids

Reports on the steroids from the genus *Litophyton* started in 1976, when two 19-hydroxysterols were reported from *L. viridis* by Bortolotto et al. [55]. Till now, 59 steroids had been obtained from six species, including *L. viridis*, *Litophyton mollis*, *L. arboreum*, *N. columnaris*, *N. erecta*, *N. chabrolii*, and unclearly identified *Litophyton* sp. and *Nephthea* sp. Structurally, 4*α*-methylated, ergostane-, cholestane-, and pregnane-type steroids dominated the steroidal profiling of this genus, with a few exceptions. The exceptional cases include one stigmastane steroid, one 13,14-*seco* steroid, one 4*α*,23-dimethylated ergostane steroid, and one rearranged steroid (Table S1). Considering their possible biogenetical relationships, the following presentation of steroids was divided into four major categories: 4*α*-methylated, ergostane, cholestane, pregnane, and their related steroids.

### 9.1. 4α-Methylated Steroids

**Figure 29:**
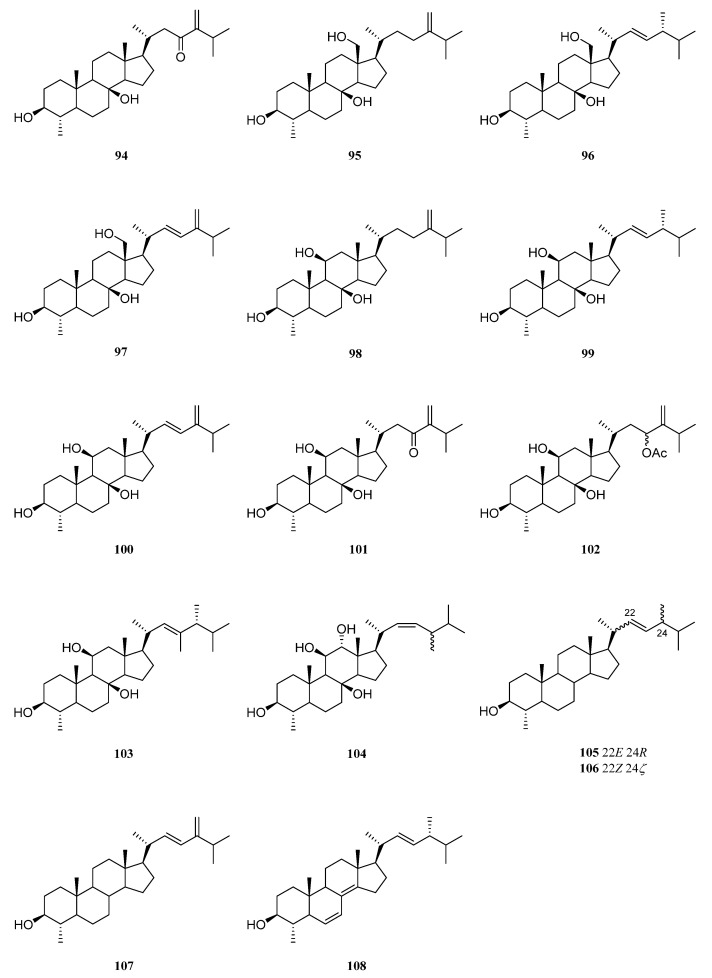
Chemical structures of the 4α-methylated steroids from soft corals of the genus *Litophyton*.

Examination of less polar fractions of the extract of the Indonesian soft coral *L. viridis*, which was collected in the Lesser Sunda Islands, led to the isolation of a novel polyoxygenated sterol 4*α*-methyl-3*β*,8*β*-dihydroxy-5*α*-ergost-24(28)-en-23-one (**94**) [56] (Figure 29). The structure and relative configuration of **94** were established unambiguously by X-ray diffraction analysis on its *p*-bromobenzoate derivative. This steroid was also obtained from Bornean octocoral *Nephthea* sp. [42]. Biological evaluation revealed compound **94** did not exhibit repellent activity against the maize weevil *S. zeamais* at 250 μg/cm^2^.

Končić et al. conducted the first chemical investigation on the metabolic profile of the Red Sea alcyonarian *L. mollis*, resulting in the isolation of ten 4*α*-methylated steroids **95**–**104** [57] (Figure 29). These steroids differed not only in the substitution of hydroxyl groups at the steroidal nucleus but also in diverse oxidation of side chains. The absolute configuration of C-24 in compounds **96**, **99**, and **103** was assigned as *R* based on the chemical shift difference between C-26 and C-27 carbon atoms, which was a powerful rule to determine the absolute configuration of steroidal side chains [58,59,60]. The cytotoxic activities of metabolites **95**–**103** were evaluated against cell lines K562 and A549 [57]. As a result, compounds **95** and **99**–**102** displayed potent cytotoxicity against K562 cells with IC_50_ values ranging from 5.6 to 8.9 μM. Meanwhile, these compounds showed low toxicity against healthy PBMCs, thus denoting interesting differential toxicity. Additionally, the tested steroids exhibited moderate levels of toxicity against A549 cells with IC_50_ values around 20 μM, further underlining their antileukemic activity.

Almost at the same time, sterol **96** was reported as a new compound from the Red Sea octocoral *Nephthea* sp., together with its analogue **94** and 4*α*,24*R*-dimethyl-5*α*-cholest-22-en-3*β*-ol (**105**) [38] (Figure 29). These three metabolites showed cytotoxicity against the cell line MCF-7 (IC_50_ 124.3, 113.6 and 201.7 μg/mL, respectively). Further study indicated the gastroprotective potential of **96** in ethanol-induced gastric ulcers in rats [61].

The Red Sea soft coral *L. arboreum* was frequently encountered by marine natural product chemists. Shaker et al. found that the Egyptian specimen *L. arboreum* harbored 4*α*,24-dimethyl-cholest-22*Z*-en-3*β*-ol (**106**) (Figure 29), the complete assignments of ^13^C NMR data of which was reported for the first time [62]. Interestingly, the presence of nebrosteroid M (**98**) in another Egyptian sample of *L. arboreum* had been reported by Mahmoud et al., which was collected in front of the National Institute of Oceanography and Fisheries at Hurghada province [37]. It was also found that sterol **98** showed cytotoxic effect against A549 cell line (IC_50_ 36.9 μmol/mL). Moreover, this compound exhibited moderate cytotoxicity against MCF-7 (IC_50_ 55.3 μmol/mL), but no activity against HepG2 (IC_50_ > 100 μmol/mL).

Ahmed et al. also made an Egyptian collection of *L. arboreum* from Neweba. Chemical investigation of this sample led to isolation of previously reported 4*α*-methylated steroids **98**, **99**, and **103** [33] (Figure 29). Anti-malarial activities against chloroquine-sensitive (D6) and chloroquine-resistant (W2) strains of *P. falciparum*, together with the cytotoxic effect against the Vero cell line, were evaluated for these three isolates. However, they were judged as inactive at the concentration of 4.76 mg/mL in the above-mentioned bioassays.

A new marine sterol, 4α-methylergosta-22(*E*),24(28)-dien-3*β*-ol (**107**) (Figure 29), was isolated from the Formosan octocoral *N*. *columnaris* [63]. Its analogue 4*α*-methyl-ergosta-6,8(14),22*E*-triene-3*β*-ol (**108**) (Figure 29) was obtained from the Red Sea soft coral *Nephthea* sp. [41]. In silica studies indicated that this compound was a potential SARS-CoV-2 main protease inhibitor.

### 9.2. Ergostane-Type and Related Steroids

**Figure 30:**
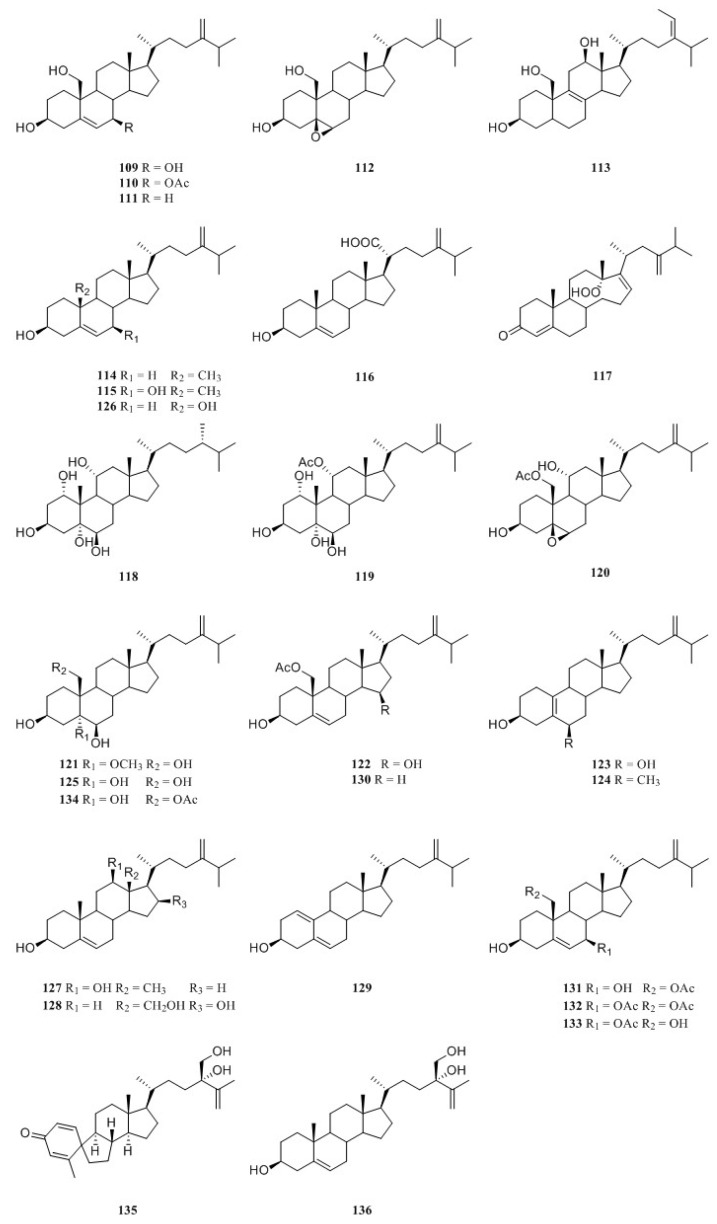
Chemical structures of the ergostane-type and related steroids from soft corals of the genus *Litophyton*.

Two novel polyhydroxylated sterols, 24-methylenecholest-5-en-3*β*,7*β*,19-triol (**109**) and its 7-monoacetate derivative (**110**) (Figure 30), were isolated from the soft coral *L*. *viridis*, collected off Serwaru, Leti Island, Maluku Province, Indonesia [55]. The structure of **109** had been established by X-ray diffraction analysis [64]. It was said these two substances were the first instances of naturally occurring 19-hydroxysterols [55]. More than ten years later, another two new 19-hydroxysterols, litosterol (**111**) and 5,6-epoxylitosterol (**112**) (Figure 30), were reported from the Okinawan sample *L*. *viridis* [65]. The latter compound showed an antileukemic activity (IC_50_ 0.5 μg/mL) against leukemia cells P388 in vitro.

Interestingly, 19-hydroxysterols **109** and **110** were widely distributed in the species *L. arboreum* collected at different waters.

Study on the substances of South China Sea alcyonarian *L. arboreum*, which was collected at Xisha Islands, led to the co-isolation of the previously reported sterol **107** and undescribed (24*E*)-24-ethyl-5*α*-cholesta-8,24(28)-diene-3*β*,12*β*,19-triol (**113**) [66] (Figure 30).

Chemical investigation of the Egyptian Red Sea soft coral *L. arboreum* by Ellithey et al., which was collected from Sharm El-Sheikh, revealed the co-existence of three steroids—**109**, **110**, and 24-methylcholesta-5,24(28)-diene-3*β*-ol (**114**) [34] (Figure 30). Compounds **109** and **110** demonstrated strong cytotoxicity against HeLa cells (IC_50_ 8 and 5.3 μM, respectively) and moderate cytotoxicity against U937 cells (IC_50_ 16.4 and 10.6 μM, respectively), whereas steroid **114** showed weak cytotoxicity against HeLa cells (IC_50_ 48 μM) and no potent cytotoxicity against U937 cells (inhibition rates < 80%). Moreover, sterol **110** displayed strong inhibitory activity against HIV-1 protease witht IC_50_ of 4.85 μM. In Ellithey’s continuous study, sterols **109** and **110** had strong cytotoxic effects against cancer cell lines HepG2 (IC_50_ 8.5 and 6.07 μg/mL, respectively), MDA (IC_50_ 5.5 and 6.3 μg/mL, respectively) and A549 (IC_50_ 9.3 and 5.2 μg/mL, respectively) [35].

Interestingly, these three sterols **82**, **83**, and **87** were also reported from the Red Sea octocoral *Nephthea* sp. [38]. These secondary metabolites were found cytotoxic against the cell line MCF-7 (IC_50_ 56.6, 37.0 and 339.2 μg/mL, respectively).

Co-existence of three known secondary metabolites **109**, **111**, and **114** in the Egyptian Red Sea collection *L. arboretum* from Hurghada was reported by Shaker et al. [62]. Recently, a study on another Egyptian Red Sea alcyonarian *L. arboreum* collected at the same coast by Mahmoud et al. disclosed the existence of sterol **114**, too [37]. In this study, metabolite **114** exhibited noticeable cytotoxicity against A549 cell line (IC_50_ 28.5 μmol/mL) and weak cytotoxic activities against both cell lines MCF-7 and HepG2 (IC_50_ 70.0 and 77.6 μmol/mL, respectively).

Chemical study of Egyptian Red Sea collection *L. arboreum* from Neweba afforded steroids **110**, **111**, 3*β*,7*β*-dihydroxy-24-methylenecholesterol (**115**), and chabrolosteroid I (**116**) [33] (Figure 30). Anti-malarial bioassays indicated that compound **115** displayed weak activity against chloroquine-resistant strain *P. falciparum* W2 with IC_50_ of 4.0 mg/mL, but was inactive against chloroquine-sensitive strain *P. falciparum* D6 at the concentration of 4.76 mg/mL.

A novel *seco*-steroid 13,14-*seco*-22-norergosta-4,24(28)-dien-19-hydroperoxide-3-one (**117**) (Figure 30) together with the known one **110** were found in the chemical investigation of Saudi Arabian Red Sea specimen *L. arboreum* by Ghandourah et al., which was collected from the North of Jeddah coast [39]. They showed antiproliferative effects toward three different cancer cell lines, MCF-7, HCT116, and HepG-2. (It might be worth pointing out no specific data were provided in this article.) In addition, Hawas et al. reported the presence of sterols **109** and **114** in another Saudi Arabian Red Sea sample *L. arboreum* [20].

Extensive studies indicated the methanolic extract of Egyptian Red Sea alcyonarian *Litophyton* sp. showed anti-colon cancer therapeutic potential. [19] The subsequent chromatography resulted in the purification of two polyhydroxylated sterols sarcsteroid F (**118**) and 24-methylenecholestane-1*α*,3*β*,5*α*,6*β*,11*α*-pentol-11-monoacetate (**119**) (Figure 30).

It might be worth pointing out that the Formosan soft corals of the title genus were abundant sources of ergostane-type and related degraded steroids.

In order to search for novel bioactive substances from Formosan soft coral *N. chabrolii*, two new 19-oxygenated steroids, nebrosteroids O and P (**120** and **121**) (Figure 30), were isolated [67]. Their cytotoxicities against A549, HT-29, and P-388 cell lines were evaluated, and the results showed they exhibited different levels cytotoxic activities with ED_50_ values ranging from 1.2 to 9.5 μg/mL. They were also examined for their antiviral activity towards human cytomegalovirus (HCMV) using a human embryonic lung (HEL) cell line. However, all of them were found to be inactive (ED_50_ > 100 μg/mL). Further chemical profiling of this specimen led to a new 19-oxygenated steroid, nebrosteroid Q (**95**) and two new cytotoxic 19-norergosterols, nebrosteroids R and S (**123** and **124**) [68] (Figure 30). Interestingly, these three sterols also displayed cytotoxic activities against A549, HT-29, and P-388 cell lines, but none of them was found to have anti-HCMV activity.

Chemical investigation of the Formosan octocoral *N. columnaris* yielded the polyhydroxyl sterol nephalsterol A (**125**) [45] (Figure 30). This metabolite **125** was found to exhibit cytotoxicities toward a panel of tumor cell lines, including MOLT-4, SUP-T1, U-937, DLD-1, LNCaP, and MCF7 with IC_50_ values of 22.5, 32.4, 38.6, 44.2, 11.6, and 9.8 μM, respectively. This naturally occurring marine steroid was synthesized and characterized as a novel neuroprotectant through negative modulation of NMDA receptors [69]. Recently, it was reported that its synthetic neuroactive derivative 5*α*-androst-3*β*,5*α*,6*β*-triol protected retinal ganglion cells from ischemia–reperfusion injury by activating the Nrf2 pathway [70].

Columnaristerol A (**126**) (Figure 30), a rare natural 19-norergostane sterol possessing a 10*β*-hydroxy group, was isolated from another Formosan octocoral *N*. *columnaris* [71]. Compound **126** might be derived from 24-methylenecholesterol (**114**) through oxidation and decarboxylation. Based on the biosynthetic derivation, the absolute configurations for the chiral carbons of **126** were assigned as 3*S*,8*S*,9*S*,10*S*,13*R*,14*S*,17*R*,20*R*. The cytotoxic effects of secondary metabolite **126** against the cell proliferation of a panel of human leukemia–lymphoma cell lines, including K-562, MOLT-4, SUP-T1, and U-937, were tested. The results revealed that **126** possessed moderate cytotoxic effects towards MOLT-4 and SUP-T1 cells (IC_50_ 18.3 and 25.5 μM, respectively). Further study on this species disclosed the presence of two new sterols, columnaristerols B (**127**) and C (**128**), along with two previously reported analogues, litosterol (**111**) and 5,6-epoxylitosterol (**112**) [72] (Figure 30). In vitro anti-inflammatory activity assays revealed that sterol **85** had inhibitory effects on the generation of superoxide anions and the release of elastase, with IC_50_ values of 4.60 and 3.90 μM, respectively.

A new 10-demethylated steroid, nephtheasteroid A (**129**), a new 19-oxygenated steroid, nephtheasteroid B (**130**), as well as five known steroids **114** and **131**–**134** (Figure 30) were isolated from the organic extract of a Formosan soft coral *N*. *erecta* [73]. The cytotoxicity of these isolates against the proliferation of a limited panel of cancer cell lines, including K562, Molt-4, Sup-T1, and U937, was evaluated. As a result, compounds **131**–**133** exhibited cytotoxicity against all or part of the above cell lines with IC_50_ values ranging from 6.5 to 19.9 μM. Further study indicated sterol **133** inhibited human small cell lung cancer growth via apoptosis induction [74].

Chabrolosteroid C (**135**), a steroid with a unique spirocyclic carbon skeleton, was identified from the Red Sea soft coral *Nephthea* sp., together with nebrosteroid O (**120**) and ergost-5,25-diene-3*β*,24*S*,28-triol (**136**) [41] (Figure 30). In silica study indicated these compounds were potential SARS-CoV-2 main protease inhibitors.

### 9.3. Cholestane-Type and Related Steroids

**Figure 31:**
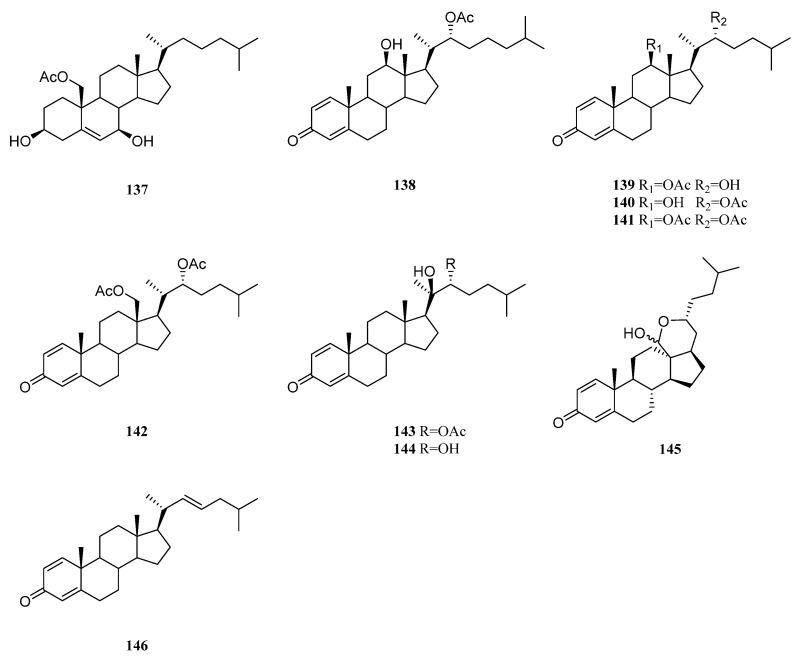
Chemical structures of the cholestane-type and related steroids from soft corals of the genus *Litophyton*.

A new 19-oxygenated steroid nebrosteroid N (**137**) (Figure 31) was isolated from Formosan soft coral *N. chabrolii* [67]. This sterol exhibited cytotoxicities against A549, HT-29, and P-388 cell lines with ED_50_ values of 6.7, 9.5, 0.9 μg/mL, respectively. However, it did not show anti-HCMV activity (ED_50_ > 100 μg/mL).

A new steroid possessing an *α*,*β*-*α*′,*β*′-unsaturated carbonyl moiety was identified in the South China Sea alcyonarian *Nephthea* sp., which was established as (20*S*,22*R*)-progesterone-1,4-diene-22-acetyl-3-one (**138**) [75] (Figure 31). This compound displayed weak cytotoxicities against A549 and Hepg2 cell lines. Further study on this species yielded six more cholesta-1,4-dien-3-one derivatives; **139**–**144** were found [76] (Figure 31). The absolute configuration at C-22 of **139** was determined to be *R* by Mosher’s method. All isolated compounds exhibited cytotoxic activity against HeLa cells with IC_50_ values ranging from 7.51 to 18.72 μg/mL. Interestingly, the existence of a novel unusual pentacyclic hemiacetal sterol nephthoacetal (**145**) (Figure 31) in the soft coral *Nephthea* sp. was disclosed [77]. Compound **145** not only strongly inhibited the settlement of *Bugula neritina* larvae with an EC_50_ value of 2.5 μg/mL, but also exhibited low toxicity towards this species of larvae with LC_50_ > 25.0 μg/mL. Moreover, this sterol showed moderate cytotoxicity against HeLa cells with an EC_50_ value of 12.3 μg/mL. Recently, dendronesterone C (**146**) (Figure 31) was obtained from the soft coral *Nephthea* sp., which were collected from the Egyptian coasts of the Red Sea at Sharm El-Sheikh [41]. In silica study indicated this compound was a potential SARS-CoV-2 main protease inhibitor.

### 9.4. Pregnane-Type and Related Steroids

**Figure 32:**
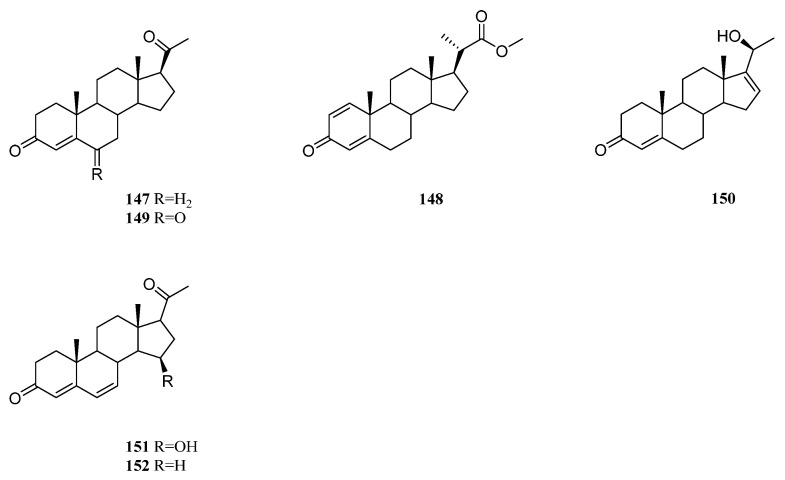
Chemical structures of the pregnane-type and related steroids from soft corals of the genus *Litophyton*.

Chemical profiling of a *Nephthea* sp. soft coral yielded six pregnane steroids, including (17*α*)-pregnan-4-ene-3,20-dione (**147**), (20*S*)-pregnan-1,4-diene-3-oxo-20-carboxylic acid methyl ester (**148**), pregnan-4-ene-3,6,20-trione (**149**), (20*R*)-pregnan-4,16-dien-20-hydroxy-3-one (**150**), pregnan-15*β*-hydroxy-4,6-diene-3,20-dione (**151**), and pregnan-4,6-diene-3,20-dione (**152**) [78] (Figure 32).

## 10. Nitrogen-Containing Metabolites

Nitrogen-containing metabolites were a small cluster of secondary metabolites from the genus *Litophyton*. This cluster consisted of 11 compounds, which could be divided into three subgroups: ceramides, pyrimidines, and peptides. These secondary metabolites were isolated from the species *L. arboretum* and *Nephthea* sp., which live in different regions of Red Sea and South China Sea (Table S1).

### 10.1. Ceramides

**Figure 33:**
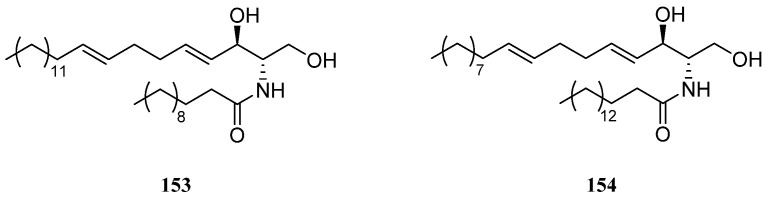
Chemical structures of the ceramides from soft corals of the genus *Litophyton*.

Chemical investigation of Red Sea alcyonarian *L. arboreum* from Sharm El-Sheikh, Egypt, afforded *erythro*-*N*-dodecanoyl-docosasphinga-(4*E*,8*E*)-dienine (**153**) (Figure 33) [34]. This metabolite showed strong inhibitory activity against HIV-1 protease (IC_50_ 4.80 μM) but exhibited weak cytotoxicity against the HeLa cell line (IC_50_ 38.17 μM). Additionally, the wide distribution of ceramide **153** in different collections of *L. arboreum* from Jeddah, Saudi Arabia [36,39], and Neweba, Egypt [33] was disclosed in these studies. It was also found in the octocoral *Nephthea* sp., which was collected from the Egyptian Red Sea off the coast of Hurghada [38]. Moreover, this metabolite **153** showed cytotoxicity against MCF-7 cell line (IC_50_ 238.5 μg/mL). However, the chemical investigation of the sample *L. arboreum* from Hurghada, yielded a different ceramide, *erythro*-*N*-palmityl-octadecasphinga-4(*E*),8(*E*)-dienine (**154**) [62] (Figure 33). This ceramide was also reported in the soft coral *Nephthea* sp., which were collected from the Egyptian coasts of the Red Sea at Sharm El-Sheikh [41]. In silica study indicated this compound was a potential SARS-CoV-2 main protease inhibitor.

### 10.2. Pyrimidines

**Figure 34:**
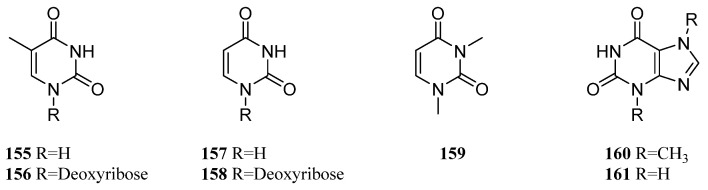
Chemical structures of the pyrimidines from soft corals of the genus *Litophyton*.

Study on the chemical constituents of Saudi Arabian soft coral *L. arboreum* led to the isolation and identification of thymine (**155**) and thymidine (**156**) [36] (Figure 34). Investigation on the compositions of Egyptian collection *L. arboreum* revealed the co-isolation of thymine (**155**), uracil (**157**), and uridine (**158**) [79] (Figure 34). Metabolites **155**, **157**, and **158** were in vitro estimated for their cytotoxic activities against three human cancer cell lines, A549, MCF-7, and HepG2, and antileishmanial potential against *Leishmania major*. However, none of them was active in these bioassays.

The existence of compounds **156** and **157** in the Chinese soft coral *Nephthea* sp. was disclosed in Xu et al.’s study [80], which were co-isolated with 1,3-dimethly uracil (**159**), caffeine (**160**), and theophylline (**161**) (Figure 34).

### 10.3. Peptides

**Figure 35:**
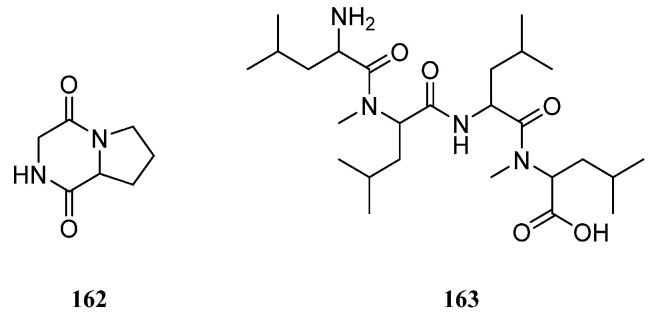
Chemical structures of the peptides from soft corals of the genus *Litophyton*.

A specimen *Nephthea* sp. was collected off the coast of Naozhou Island, South China Sea, which afforded a cyclic peptide named cyclo-(Pro-Gly) (**162**) [80] (Figure 35). Further chemical investigation of this species led to a new tetrapeptide, which was established as leucyl-*N*-methyl-leucyl-leucyl-*N*-methyl-leucine (**163**) [75] (Figure 35). This compound showed weak cytotoxicities against A549 and Hepg2 cell lines.

## 11. Lipids

This cluster consisted of one prostaglandin, four *γ*-lactones, four fatty acids, and two glycerol ethers. These secondary metabolites distributed in *L. arboretum* and unclearly identified *Litophyto*n sp. which were collected in the Red Sea and the waters around Japan (Table S1).

### 11.1. Prostaglandins

**Figure 36:**
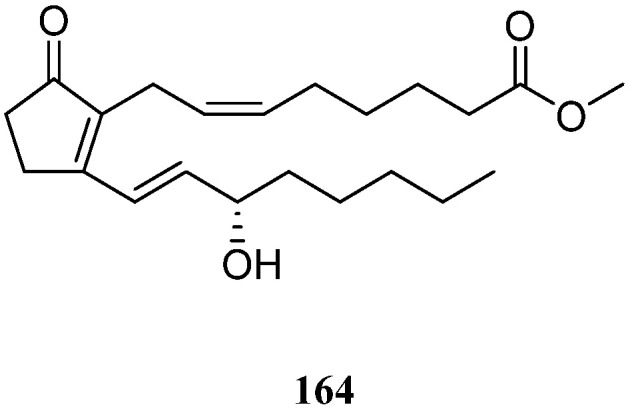
Chemical structure of the prostaglandin from soft corals of the genus *Litophyton*.

The sole prostaglandin from the genus *Litophyton*, PGB_2_ methyl ester (**164**) (Figure 36), was characterized in the research of Red Sea alcyonarian *L. arboreum*, which lived in the gulf of Aqaba, Eilat, Israel [81].

### 11.2. γ-Lactones

**Figure 37:**
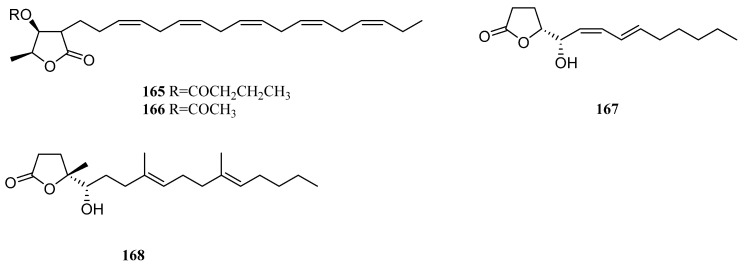
Chemical structures of the *γ*-lactones from soft corals of the genus *Litophyton*.

Two new branched-chain lipids containing a *γ*-lactone ring, which was named litophytolides A (**165**) and B (**166**) (Figure 37), were isolated from a Japanese soft coral *Litophyton* sp. [82]. The difference in their structures was the replacement of the butyryl group in **165** by the acetyl group in **166**.

Chemical study of Israeli Red Sea alcyonarian *L. arboreum* led to the discovery of two novel *γ*-lactones with unsaturated chains **167** and **168** [81] (Figure 37). The absolute configuration of C-5 was assigned as *S* for **167** and **168** by applying the Mosher’s method. In the toxicity bioassay, these two secondary metabolites were toxic to brine shrimp *Artemia salina* (CC_50_ 15.3 and 21.4 μg/mL, respectively). Antibacterial evaluations indicated the two *γ*-lactones were active only against Gram-positive bacteria *S. aureus* and *B. subtilis* with diameters of inhibition zones ranging from 5.6 to 18.6 mm, but they were inactive against Gram-negative bacterium *E. coli* and yeast *Saccharomyces cerevisiae*.

### 11.3. Fatty Acids

**Figure 38:**
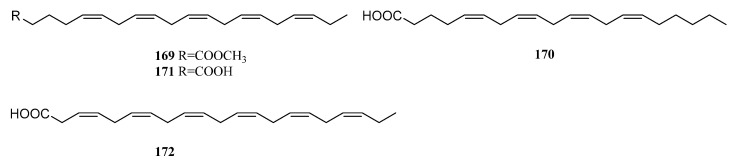
Chemical structures of the fatty acids from soft corals of the genus *Litophyton*.

During a search for the chemical constituents of a Japanese soft coral *Litophyto*n sp., methyl (5*Z*,8*Z*,11*Z*,14*Z*,17*Z*)-5,8,11,14,17-icosapentaenoate (**169**) was encountered, together with the above-described *γ*-lactones litophytolides A (**165**) and B (**166**) [82] (Figure 38). The co-occurrence of litophytolides **165** and **166** and unsaturated fatty acid **169** in the same animal led to the proposed biogenesis of branched-chain lipids with a *γ*-lactone ring that involved the condensation of unsaturated fatty acids with pyruvate. GC-MS analysis of the fraction of a Israeli alcyonarian *L. arboreum* revealed the presence of arachidonic acid (**170**), eicosapentaenoic acid (**171**) and docosahexaenoic acid (**172**) [81] (Figure 38).

### 11.4. Glycerol Ethers

**Figure 39:**
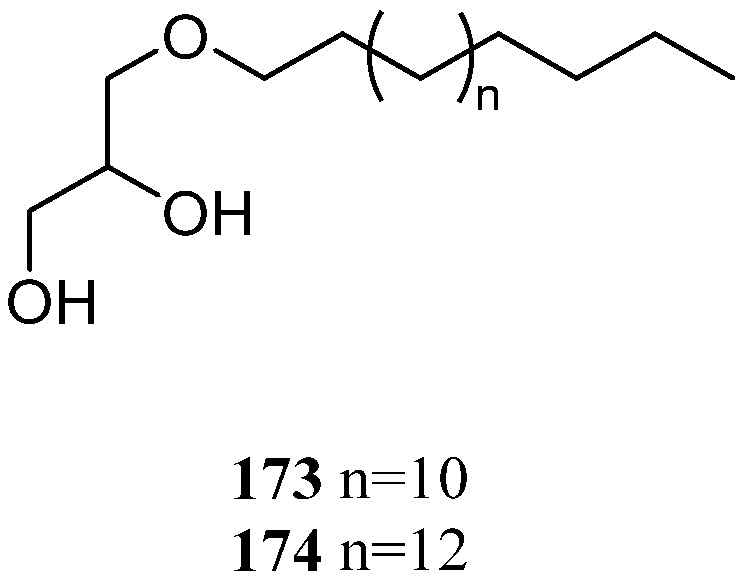
Chemical structures of the glycerol ethers from soft corals of the genus *Litophyton*.

Investigation of the chemical compositions of Red Sea soft coral *L. arboreum*, which inhabited the coast of Sharm El-Sheikh, Egypt, resulted in the isolation and characterization of chimyl alcohol (**173**) [34] (Figure 39). This alcohol not only showed cytotoxic effects against the cell lines HeLa and Vero (IC_50_ 23.35 and 60 μM, respectively), but also exhibited inhibitory activity against HIV-1 protease (IC_50_ 26.6 μM). The presence of **173** in the Saudi Arabian Red Sea sample *L. arboreum* was reported [36].

Chemical study of Red Sea specimen *L. arboreum*, which was collected at Hurghada, Egypt, disclosed the co-existence of chimyl alcohol (**173**) and batyl alcohol (**174**) [37] (Figure 39). Cytotoxicity bioassays were also performed for these two glycerol ethers, but none of them was active against the tested cell lines A549, MCF-7, and HepG2 (all IC_50_ > 100 µmol/mL).

## 12. Other Metabolites

**Figure 40:**
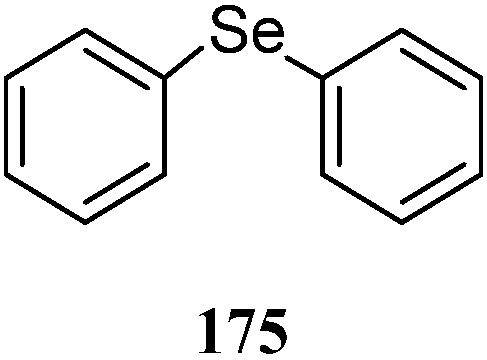
Chemical structure of the selenide from soft corals of the genus *Litophyton*.

Interestingly, diphenyl selenide (**175**) (Figure 40) was identified as a secondary metabolite of a soft coral *Nephthea* sp., which resided in South China Sea [80].

## 13. Discussion

**Figure 41:**
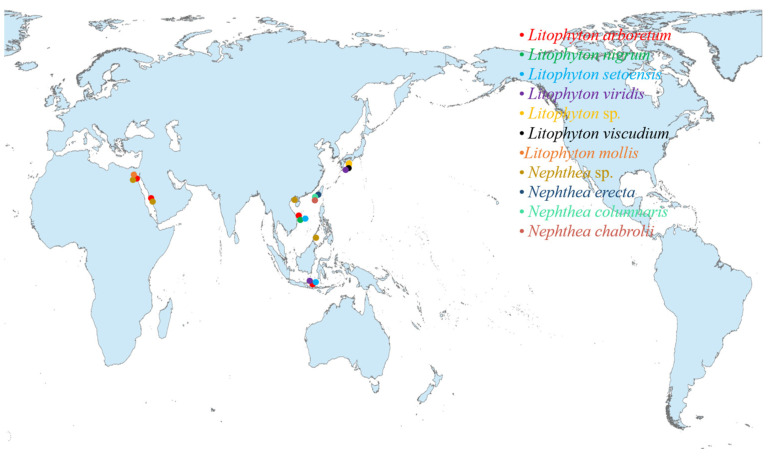
The distribution of the investigated species of the genus *Litophyton*.

The current work presents an up-to-date documentation of the reported studies on the genus *Litophyton* with a special focus on their diverse chemical classes of secondary metabolites and their bioactivities. The investigated soft corals of this genus inhabited various marine environments from tropical to temperate regions, especially in the South China Sea, Red Sea, and the waters around Taiwan, Indonesia, Malaysia, and Japan (Figure 41, Table S1).

**Figure 42:**
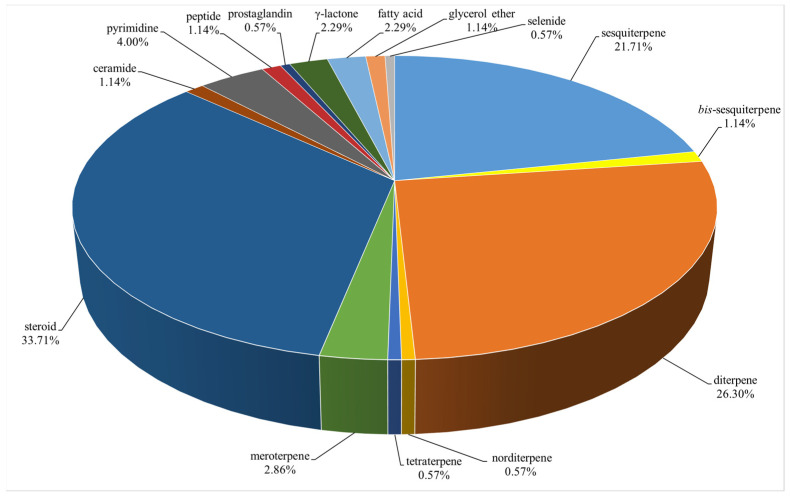
Chemical profile of secondary metabolites from soft corals of the genus *Litophyton*.

A total of 175 compounds from a variety of species of this genus were reported from 1975 to July 2023, covering a period of almost five decades. These substances illustrated in this work could be categorized as sesquiterpenes, sesquiterpene dimers, diterpenes, norditerpenes, tetraterpenes, meroterpenes, steroids, ceramides, pyrimidines, peptides, prostaglandins, *γ*-lactones, fatty acids, glycerol ethers, and selenides (Figure 42), most of which could be grouped in four major chemical classes: terpenoids, steroids, nitrogen-containing metabolites, and lipids. Among them, terpenes were predominant chemical constituents accounting for 53.14%, which consisted of 38 sesquiterpenes (21.71%), 2 *bi*s-sesquiterpenes (1.14%), 46 diterpenes (26.29%), 1 norditerpene (0.57%), 1 tetraterpene (0.57%), and 5 meroterpenes (2.86%) (Figure 42, Table S1). Additionally, the very recently reported one s*ec*-germacrane sesquiterpene [33], one himachalene sesquiterpene [39], one nornardosinane sesquiterpene [14], one seconeolemnane sesquiterpene [14], one kelsoane sesquiterpene [30], one *bis*-kelsoane *bis*-sesquiterpene [30], one eremophilane-nardosinane *bis*-sesquiterpene [31], and five 5,9-cyclized serrulatane diterpenes [16] were quite uncommon marine natural products, some of which were only identified in the genus *Litophyton*.

**Figure 43:**
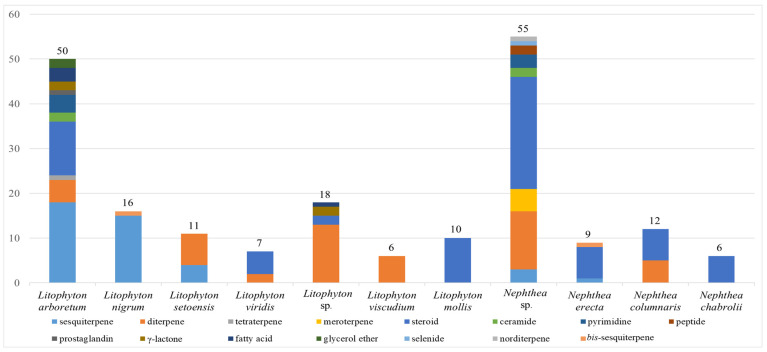
Number of compounds reported from different species of the genus *Litophyton*.

Chemical investigations have been conducted on the species *Litophyton arboreum*, *Litophyton nigrum*, *Litophyton setoensis*, *Litophyton viridis*, *Litophyton viscudium*, *Litophyton mollis*, *Nephthea erecta*, *Nephthea columnaris*, *Nephthea chablrolii*, and unclearly identified *Litophyton* sp. and *Nephthea* sp. In terms of the numbers of isolated substances, *L. arboreum* was the most frequently studied species of this genus, yielding 50 compounds (Figure 43). The metabolites of *L. arboreum* comprised almost all structural types of chemical compositions from the title genus, including 18 sesquiterpenes, 5 diterpenes, 1 tetraterpene, 12 steroids, 2 ceramides, 4 pyrimidines, 1 prostaglandin, 2 *γ*-lactones, 3 fatty acids, and 2 glycerol ethers (Figure 43, Table S1). Interestingly, bicyclogermacrane, s*ec*-germacrane, guaiane, pseudoguaiane, himachalene, eudesmane, *seco*-eudesmane, and tri-nor-eudesmane sesquiterpenes were only isolated and characterized from the alcyonarian *L. arboreum*, which could be regarded as a chemotaxonomic marker for this species (Table S1). Similarly, eremophilane, nornardosinane, and seconeolemnane sesquiterpenes, especially an eremophilane-nardosinane *bis*-sesquiterpene, could provide the chemotaxonomic evidence for the species *L. nigrum* (Table S1). Meanwhile, the chemotaxonomic characters for the species *L. setoensis* were serrulatane and 5,9-cyclized serrulatane diterpenes, for *N. erecta* were kelsoane sesquiterpene and its dimer, and for *N*. *chablrolii* was chabrolane diterpene (Table S1).

As summarized in this review, the species of the original genera *Litophyton* and *Nephthea* shared a number of secondary metabolites with various structural features (e.g., guaiane sesquiterpenes 10-*O*-methyl alismoxide (**4**) and alismoxide (**5**) for *L. arboreum* and *Nephthea* sp., cembrane diterpenes nephtenol (**41**) and 2-hydroxynephtenol (**42**) for *L. viridis* and *Nephthea* sp., eunicellane diterpene litophynin C for *Litophyton* sp. and *Nephthea* sp., 4α-methylated steroid 4*α*-methyl-3*β*,8*β*-dihydroxy-5*α*-ergost-24(28)-en-23-one (**94**) and ergostane steroid 24-methylenecholest-5-en-3*β*,7*β*,19-triol (**109**) for *L. viridis* and *Nephthea* sp., ceramides *erythro*-*N*-dodecanoyl-docosasphinga-(4*E*,8*E*)-dienine (**153**) and *erythro*-*N*-palmityl-octadecasphinga-4(*E*),8(*E*)-dienine (**154**) for *L. arboreum* and *Nephthea* sp., etc.) (Table S1). These might be served as compelling evidence for the taxonomic consolidation of the genera *Nephthea* and *Litophyton* into the genus *Litophyton* from the view of the natural products.

These metabolites exhibited a wide spectrum of bioactivities including cytotoxic, anti-malarial, antibacterial, antifungal, antiviral, antifeedant, molluscicidal, PTP1B inhibitory, insect growth inhibitory, and neuroprotective effects (Table S1). The most frequently evaluated activity for the chemical constituents of the genus *Litophyton* was cytotoxicity against a panel of human cancer cell lines, such as HeLa, K-562, HepG2, MDA, A549, MCF-7, HCT116, U937, SUN-398, HT-29, Capan-1, THP-1, HL-60, P388, MOLT-4, SUP-T1, DLD-1, and LNCaP, and quite a high number of compounds showed growth inhibitory activities against some of the above-mentioned cell lines. Interestingly, nephalsterol A (**125**) was found to be not only a cytotoxic agent toward a panel of tumor cell lines, including MOLT-4, SUP-T1, U-937, DLD-1, LNCaP, and MCF7 with IC_50_ values of 22.5, 32.4, 38.6, 44.2, 11.6, and 9.8 μM, respectively [45], but also a potent neuroprotectant through negative modulation of NMDA receptors [69]. Recently, its synthetic derivative 5*α*-androst-3*β*,5*α*,6*β*-triol was also a neuroactive substance that protected retinal ganglion cells from ischemia–reperfusion injury by activating the Nrf2 pathway [70]. It might be worth pointing out that although the molluscicidal activity against the muricid gastropod *D. fragum* [52] and toxic activity to brine shrimp *A. salina* [81] were performed respectively for eunicellane diterpenes and *γ*-lactones, more research should be conducted to better understand the ecological roles of *Litophyton* secondary metabolites.

## 14. Conclusions

As presented in this work, the soft corals of the genus *Litophyton* harbor an array of structurally unique and diversely bioactive secondary metabolites, including sesquiterpenes, sesquiterpene dimers, diterpenes, norditerpenes, tetraterpenes, meroterpenes, steroids, ceramides, pyrimidines, peptides, prostaglandins, γ-lactones, fatty acids, glycerol ethers, and selenides (Figure 42, Table S1). However, only nine species including *L. arboreum*, *L. nigrum*, *L. setoensis*, *L. viridis*, *L. viscudium*, *L. mollis*, *N. erecta*, *N. columnaris*, and *N. chablrolii* have been investigated besides unclearly identified *Litophyton* sp. and *Nephthea* sp. (Figure 43, Table S1), which were a very small proportion of the whole genus *Litophyton* [10]. It is clear that there is a need for increased research on exploration of more species of this genus, which are hidden treasure troves of novel marine natural products.

As shown in this work, the eunicellane and cembrene diterpenes displayed a broad range of bioactivities including antifungal, anti-HIV, antitumor, anti-inflammatory, antibacterial, insect growth inhibitory, and molluscicidal properties (Table S1). However, due to the limited amounts of these metabolites in soft corals, exploration of new technologies to gain efficient substances is becoming impendingly demanded. Very recently, terpene cyclase genes that produce eunicellane and cembrene diterpenes have been found in soft corals such as *Erythropodium caribaeorum* and *Dendronephtha gigantean* [83,84]. Investigating the biogenesis of the secondary metabolites of the genus *Litophyton* and the utilization of biosyntheses in rapid production of terpenes would be another significant and hot research topic in this field. Moreover, the discovery of novel terpene biosynthetic gene clusters could provide potential bioengineering applications for industry. 

## Data Availability

Not applicable.

## Supplementary Materials

The following supporting information can be downloaded at: https://www.mdpi.com/article/10.3390/md21100523/s1, Table S1: Secondary metabolites of the genus *Litophyton* from 1975 to July 2023.
