# Supplementary material for: Genus Litophyton: A Hidden Treasure Trove of Structurally Unique and Diversely Bioactive Secondary Metabolites
Source: Mar Drugs. 2023 Sep 29;21(10):523. doi: 10.3390/md21100523 (PMC10608288; doi:10.3390/md21100523)
Supplement: Supplementary file 1 [file marinedrugs-21-00523-s001.zip › marinedrugs-2610574-Supplementary Materials_proof.pdf]

Supplementary Materials

Table S1. Secondary metabolites of the genus *Litophyton* from 1975 to July, 2023.

No.	Name	Class	Species	Locality	Bioassays	Ref.
**1**	(–)-bicyclogermacrene	bicyclogermacrane sesquiterpene	*Litophyton arboreum*	near Bali, Indonesia	antiproliferative activities against the L-929 and K-562 cell lines (GI_{50} 186±6 and 200±8 μM, respectively), cytotoxic effect against the HeLa cell line (CC_{50} 182±8 μM)	[37]
**2**	(2*E*,6*E*)-3-isopropyl-6-methyl-10-oxoundeca-2,6-dienal	*sec*-germacrane sesquiterpene	*L. arboreum*	Neweba, Egypt (Red Sea)	anti-malarial activity against chloroquine-sensitive *Plasmodium falciparum* D6 and chloroquine-resistant *Plasmodium falciparum* W2 (IC_{50} 3.7 amd 2.2 mg/mL, respectively), non-toxic to the Vero cell line at the concentration of 4.76 mg/mL	[19]
**3**	alismol	guaiane sesquiterpene	*L. arboreum*	Sharm El-Sheikh, Egypt (Red Sea)	inhibitory activity against HIV-1 protease receptor (IC_{50} 7.20±0.7 μM), cytotoxic activities against the HeLa and Vero cell lines (IC_{50} 30 and 49 μM, respectively)	[38]
			L. arboreum	Sharm El-Sheikh, Egypt (Red Sea)	cytotoxic effects against the HepG2, MDA and A549 cancer cell lines (IC_{50} 4.52, 7.02 and 9.23 μg/mL, respectively)	[39]
			L. arboreum	Jeddah, Saudi Arabia (Red Sea)	antibacterial activities against *Escherichia coli* ATCC 10536, *Pseudomonas aeruginosa* NTCC 6750, *Bacillus cereus* ATCC 9634, *Bacillus subtilis* ATCC6633, *Staphylococcus aureus* ATCC5141 (MIC 9.2, 10.4, 4.3, 5.0, and 9.2 μg/mL, respectively), antifungal activities against *Candida albicans* and *Aspergillus niger* (MIC 10.1 and 7.2 μg/mL, respectively), cytotoxic effects against the MCF-7, HCT-116 and HepG2 cell lines (IC_{50} 44.52±0.5, 31.45±1.19, and 10.71±1.18 μM, respectively)	[40]
			L. arboreum	Neweba, Egypt (Red Sea)	[a] –	[19]
**4**	10-*O*-methyl alismoxide	guaiane sesquiterpene	*L. arboreum*	Sharm El-Sheikh, Egypt (Red Sea)	cytotoxic activities against the HeLa, Vero and U937 cell lines (IC_{50} 38±0.7, 49.8±0.5 and 50±0.23 μM, respectively)	[38]

No.	Name	Class	Species	Locality	Bioassays	Ref.
			Nephthea sp.	Hurghada, Egypt (Red Sea)	cytotoxicity against the MCF-7 cell line (IC_{50} 85.5 μg/mL)	[42]
**5**	alismoxide	guaiane sesquiterpene	*L. arboreum*	Sharm El-Sheikh, Egypt (Red Sea)	inactive against the HeLa, Vero and U937 cell lines (all IC_{50} >100 μM)	[38]
			L. arboreum	Hurghada, Egypt (Red Sea)	inactive against the A549, MCF-7 and HepG2 cell lines (all IC_{50} >100 μmol/mL)	[41]
			L. arboreum	Neweba, Egypt (Red Sea)	[a] –	[19]
			Nephthea sp.	Hurghada, Egypt (Red Sea)	cytotoxicity against the MCF-7 cell line (IC_{50} 151.9 μg/mL)	[42]
**6**	alismorientol B	guaiane sesquiterpene	*L. arboreum*	Jeddah, Saudi Arabia (Red Sea)	antibacterial activities against *E. coli* ATCC 10536, *P. aeruginosa* NTCC 6750, *B. cereus* ATCC 9634, *B. subtilis* ATCC6633, *S. aureus* ATCC5141 (MIC 3.4, 7.7, 1.3, 6.8, and 8.3 μg/mL, respectively), antifungal activities against *C. albicans* and *A. niger* (MIC 6.0 and 8.4 μg/mL, respectively), cytotoxic effects against the MCF-7, HCT-116 and HepG2 cell lines (IC_{50} 4.32±0.13, 27.86±1.28 and 12.27±1.09 μM, respectively)	[40]
**7**	litoarbolide A	guaiane sesquiterpene	*L. arboreum*	Neweba, Egypt (Red Sea)	no anti-malarial activity against chloroquine-sensitive *P. falciparum* D6 and chloroquine-resistant *P. falciparum* W2 at the concentration of 4.76 mg/mL, non-toxic to the Vero cell line at the concentration of 4.76 mg/mL	[19]
**8**	4α,7β,10α-trihydroxyguai-5-ene	guaiane sesquiterpene	*L. arboreum*	Neweba, Egypt (Red Sea)	no anti-malarial activity against chloroquine-sensitive *P. falciparum* D6 and chloroquine-resistant *P. falciparum* W2 at the concentration of 4.76 mg/mL, non-toxic to the Vero cell line at the concentration of 4.76 mg/mL	[19]
**9**	leptocladol B	guaiane sesquiterpene	*L. arboreum*	Neweba, Egypt (Red Sea)	anti-malarial activity against chloroquine-resistant *P. falciparum* W2 (IC_{50} 4.3 mg/mL), no anti-malarial effect againste hloroquine-sensitive *P. falciparum* D6 at the concentration of 4.76 mg/mL, non-toxic to the Vero cell line	[19]

No.	Name	Class	Species	Locality	Bioassays	Ref.
					at the concentration of 4.76 mg/mL	
**10**	nephthetetraol	guaiane sesquiterpene	*L. arboreum*	Neweba, Egypt (Red Sea)	anti-malarial activity against chloroquine-resistant *P. falciparum* W2 (IC_{50} 3.2 mg/mL), no anti-malarial effect againste hloroquine-sensitive *P. falciparum* D6 at the concentration of 4.76 mg/mL, non-toxic to the Vero cell line at the concentration of 4.76 mg/mL	[19]
**11**	litopharbol	pseudoguaiane sesquiterpene	*L. arboreum*	Jeddah, Saudi Arabia (Red Sea)	antibacterial activities against *E. coli* ATCC 10536, *P. aeruginosa* NTCC 6750, *B. cereus* ATCC 9634, *B. subtilis* ATCC6633, *S. aureus* ATCC5141 (MIC 9.0, 3.6, 1.8, 5.8, and 9.6 μg/mL, respectively), antifungal activities against *C. albicans* and *A. niger* (MIC 12.5 and 12.9 μg/mL, respectively), cytotoxic effects against the MCF-7, HCT-116 and HepG2 cell lines (IC_{50} 9.42±0.17, 26.21±2.249 and 38.92±1.12 μM, respectively)	[40]
**12**	litopharbdiol	pseudoguaiane sesquiterpene	*L. arboreum*	Jeddah, Saudi Arabia (Red Sea)	[a] –	[20]
**13**	3*α*,6*α*-epidioxyhimachal-1-ene	himachalene sesquiterpene	*L. arboreum*	Jeddah, Saudi Arabi (Red Sea)	[b] antiproliferative effects toward MCF-7, HCT116 and HepG2 cell lines	[43]
**14**	5*β*,8*β*-epidioxy-11-hydroxy-6-eudesmene	eudesmane sesquiterpene	*L. arboreum*	Hurghada, Egypt (Red Sea)	cytotoxic effect against A549 (IC_{50} 67.3±9.9 μmol/mL), inactive against the MCF-7 and HepG2 cell lines (both IC_{50} >100 μmol/mL)	[41]
**15**	chabrolidione B	*seco*-eudesmane sesquiterpene	*L. arboreum*	Hurghada, Egypt (Red Sea)	inactive against the A549, MCF-7 and HepG2 cell lines (all IC_{50} >100 μmol/mL)	[41]
**16**	teuhetenone A	tri-nor-eudesmane sesquiterpene	*L. arboreum*	Jeddah, Saudi Arabia (Red Sea)	antibacterial activities against *E. coli* ATCC 10536, *P. aeruginosa* NTCC 6750, *B. cereus* ATCC 9634, *B. subtilis* ATCC6633, *S. aureus* ATCC5141 (MIC 1.9, 2.8, 5.4, 4.3, and 10.9 μg/mL, respectively), antifungal activities against *C. albicans* and *A. niger* (MIC 4.1 and 7.4 μg/mL, respectively), cytotoxic effect against the HepG2 cell line (IC_{50} 39.23±3.21 μM), non-toxic to the MCF-7 and HCT-116 cell lines (both	[40]

No.	Name	Class	Species	Locality	Bioassays	Ref.
					IC_{50} >100 μM)	
**17**	calamusin I	tri-nor-eudesmane sesquiterpene	*L. arboreum*	Jeddah, Saudi Arabia (Red Sea)	antibacterial activities against *E. coli* ATCC 10536, *P. aeruginosa* NTCC 6750, *B. cereus* ATCC 9634, *B. subtilis* ATCC6633, *S. aureus* ATCC5141 (MIC 3.2, 1.2, 6.1, 7.8, and 7.4 μg/mL, respectively), antifungal activities against *C. albicans* and *A. niger* (MIC 3.2 and 5.6 μg/mL, respectively), cytotoxic effects against the MCF-7 and HepG2 cell lines (IC_{50} 6.43±0.23 and 13.46±1.16 μM, respectively), non-toxic to the HCT-116 cell line (IC_{50} >100 μM)	[40]
**18**	7-oxo-tri-nor-eudesm-5-en-4*β*-ol	tri-nor-eudesmane sesquiterpene	*L. arboreum*	Hurghada, Egypt (Red Sea)	inactive against the A549, MCF-7 and HepG2 cell lines (all IC_{50} >100 μmol/mL)	[41]
**19**	11,12-dihydroxy-6,10-eremophiladiene	eremophilane sesquiterpene	*Litophyton nigrum*	Xisha Islands, Hainan, China (South China Sea)	[a] –	[36]
**20**	linardosinene B	nardosinane sesquiterpenes	*L. nigrum*	Xisha Islands, Hainan, China (South China Sea)	cytotoxic effect against the THP-1 cell line (IC_{50} 59.49 μM)	[15]
**21**	linardosinene C	nardosinane sesquiterpenes	*L. nigrum*	Xisha Islands, Hainan, China (South China Sea)	cytotoxic activities against the SNU-398 and HT-29 cell lines (IC_{50} 24.3 and 44.7 μM, respectively), inactive against the Capan-1 and A549 cell lines (both IC_{50} >50 μM)	[15]
**22**	linardosinene D	nardosinane sesquiterpenes	*L. nigrum*	Xisha Islands, Hainan, China (South China Sea)	inhibitory effect against BRD4 at a concentration of 10 μM with inhibitory rate 16.3%	[44]
**23**	linardosinene E	nardosinane sesquiterpenes	*L. nigrum*	Xisha Islands, Hainan, China (South China Sea)	inhibitory effect against BRD4 at a concentration of 10 μM with inhibitory rate 15.8%	[44]
**24**	linardosinene F	nardosinane sesquiterpenes	*L. nigrum*	Xisha Islands, Hainan, China (South China Sea)	inhibitory effect against BRD4 at a concentration of 10 μM with inhibitory rate 17.6%	[44]
**25**	linardosinene G	nardosinane	*L. nigrum*	Xisha Islands, Hainan,	inhibitory effect against BRD4 at a concentration of 10 μM	[44]

No.	Name	Class	Species	Locality	Bioassays	Ref.
		sesquiterpenes		China (South China Sea)	with inhibitory rate 18.1%	
**26**	linardosinene I	nardosinane sesquiterpenes	*L. nigrum*	Xisha Islands, Hainan, China (South China Sea)	inhibitory activity against PTP1B (IC_{50} 10.67 ± 1.81 μg/mL), cytotoxic activities against the HT-29, Capan-1 and SNU-398 cell lines (IC_{50} 35.48 ± 11.22, 42.55 ± 2.51, and 25.17 ± 11.13 μM, respectively)	[36]
**27**	emnal-l(l0)-ene-7*β*,12*α*-diol	nardosinane sesquiterpenes	*L. nigrum*	Xisha Islands, Hainan, China (South China Sea)	inactive against PTP1B (IC_{50} >20 μg/mL) and the HT-29, Capan-1 and SNU-398 cell lines (all IC_{50} >50 μM)	[36]
**28**	paralemnolin J	nardosinane sesquiterpenes	*Litophyton setoensis*	Singaraja, Bali Island, Indonesia	weak cytotoxic activities against the HT-29, Capan-1, A549, and SNU-398 cell lines (all IC_{50} >20 μM)	[17]
**29**	(l*S*,8*S*,8*aS*)-*l*-[(*E*)-2′-acetoxy-l′-methylethenyl]-8,8*a*-dimethyl-3,4,6,7,8,8*a*-hexahydronaphthalen-2(1*H*)-one	nardosinane sesquiterpenes	*L. setoensis*	Singaraja, Bali Island, Indonesia	weak cytotoxic activities against the HT-29, Capan-1, A549, and SNU-398 cell lines (all IC_{50} >20 μM)	[17]
**30**	linardosinene A	nornardosinane sesquiterpenes	*L. nigrum*	Xisha Islands, Hainan, China (South China Sea)	inactive against the THP-1 cell line and the PTP1B, BRD4, HDAC1 and HDAC6 protein kinases	[15]
**31**	lineolemnene A	neolemnane sesquiterpene	*L. nigrum*	Xisha Islands, Hainan, China (South China Sea)	cytotoxic activity against the SNU-398 cell line (IC_{50} 44.4 μM), inactive against the HT-29, Capan-1 and A549 cell lines (all IC_{50} >50 μM)	[15]
**32**	lineolemnene B	neolemnane sesquiterpene	*L. nigrum*	Xisha Islands, Hainan, China (South China Sea)	cytotoxic activity against the SNU-398 cell line (IC_{50} 27.6 μM), inactive against the HT-29, Capan-1 and A549 cell lines (all IC_{50} >50 μM)	[15]
**33**	lineolemnene C	neolemnane sesquiterpene	*L. nigrum*	Xisha Islands, Hainan, China (South China Sea)	inactive against the HT-29, Capan-1, A549, and SNU-398 cell lines (all IC_{50} >50 μM)	[15]
**34**	4-acetoxy-2,8-neolemnadien-5-one	neolemnane sesquiterpene	*L. nigrum*	Xisha Islands, Hainan, China (South China Sea)	[a] –	[15]
			L. setoensis	Singaraja, Bali Island,	weak cytotoxic activities against the HT-29, Capan-1, A549,	[17]

No.	Name	Class	Species	Locality	Bioassays	Ref.
				Indonesia	and SNU-398 cell lines (all IC_{50} >20 μM)	
**35**	paralemnolin E	neolemnane sesquiterpene	*L. setoensis*	Singaraja, Bali Island, Indonesia	weak cytotoxic activities against the HT-29, Capan-1, A549, and SNU-398 cell lines (all IC_{50} >20 μM)	[17]
**36**	parathyrsoidin E	neolemnane sesquiterpene	*Nephthea* sp.	Sharm El-Sheikh, Egypt (Red Sea)	potential SARS-CoV-2 main protease inhibitor *in silica*	[45]
**37**	lineolemnene D	seconeolemnane sesquiterpene	*L. nigrum*	Xisha Islands, Hainan, China (South China Sea)	inactive against the THP-1 cell line and the PTP1B, BRD4, HDAC1 and HDAC6 protein kinases	[15]
**38**	kelsoenethiol	kelsoane sesquiterpene	*Nephthea erecta*	Green Island, Taiwan	cytotoxic activities against the P-388 and HT-29 cell lines (ED_{50} 1.3 and 1.8 μg/mL, respectively)	[22]
**39**	dikelsoenyl ether	*bis*-kelsoane *bis*-sesquiterpene	*N. erecta*	Green Island, Taiwan	inactive against the A-459, P-388 and HT-29 cell lines	[22]
**40**	linardosinene H	eremophilane-nardosinane *bis*-sesquiterpene	*L. nigrum*	Xisha Islands, Hainan, China (South China Sea)	inactive against PTP1B (IC_{50} >20 μg/mL) and the HT-29, Capan-1 and SNU-398 cell lines (all IC_{50} >50 μM)	[36]
**41**	nephtenol	cembrane diterpene	*Litophyton viridis*	Serwaru, Leti Island, Indonesia	[a] –	[21]
			L. arboreum	Hurghada, Egypt (Red Sea)	[a] –	[47]
			L. arboreum	Jeddah, Saudi Arabia (Red Sea)	[a] –	[20]
			Nephthea sp.	Mantanani Island, Sabah, Malaysia	no repellent activity against the maize weevil *Sitophilus zeamais* at 250 μg/cm^2	[46]
**42**	2-hydroxynephtenol	cembrane diterpene	*L. viridis*	Serwaru, Leti Island, Indonesia	[a] –	[21]
			Nephthea sp.	Mantanani Island, Sabah, Malaysia	no repellent activity against the maize weevil *S. zeamais* at 250 μg/cm^2	[46]
			Nephthea sp.	Sepanggar Bay, Sabah, North Borneo, Malaysia	antifungal activities against *Exophiala* sp. NJM 1551, *Fusarium moniliforme* NJM 8995, *Fusarium oxysporum* NJM 0179, *Fusarium solani* NJM 8996, *Haliphthoros sabahensis*	[25]

No.	Name	Class	Species	Locality	Bioassays	Ref.
					IPMB 1402, *Haliphthoros milfordensis* IPMB 1603, and *Lagenidium thermophilum* IPMB 1401 (MIC 25, 50, 50, 50, 25, 25, and 12.5 μg/mL, respectively)	
**43**	(3*E*,11*E*)-cembra-3,8(19),11,15-tetraene-7*α*-ol	cembrane diterpene	*L. arboreum*	Hurghada, Egypt (Red Sea)	[a] –	[47]
**44**	sarcophytol M	cembrane diterpene	*L. arboreum*	Sharm El-Sheikh, Egypt (Red Sea)	inhibitory activity against HIV-1 protease receptor (IC_{50} 15.7±0.10 μM), cytotoxic activities against the HeLa, Vero and U937 cell lines (IC_{50} 27.5±0.2, 22±0.2 and 31.7±3.2 μM, respectively)	[38]
**45**	11-acetoxy-15,17-dihydroxy-2,12-epoxy-(3*E*,7*E*)-1-cembra-3,7-diene	cembrane diterpene	*L. arboreum*	Jeddah, Saudi Arabia (Red Sea)	antiproliferative effects toward the MCF-7, HCT116 and HepG2 cell lines (IC_{50} 19.1±0.032, 22.0±0.092, 24.0±0.032 μM, respectively)	[43]
**46**	11*β*,12*β*-epoxypukalide	cembrane diterpene	*L. arboreum*	near Bali, Indonesia	antiproliferative activities against the L-929 and K-5629 cell lines (both GI_{50} >129 μM, respectively), cytotoxic effect against the HeLa cell line (CC_{50} 115±6 μM)	[37]
**47**	columnariol A	cembrane diterpene	*Nephthea columnaris*	Southern Taiwan	significantly inhibit the accumulation of the pro-inflammatory iNOS and COX-2 protein of the lipopolysaccharide (LPS)-stimulated RAW264.7 macrophage cells; moderate cytotoxicity against the LNCaP cell line (IC_{50} 9.80 μg/mL), inactive against the MOLT-4, SUP-T1, U-937, DLD-1, and MCF7 cell lines at 20 μg/mL	[23]
**48**	columnariol B	cembrane diterpene	*N. columnaris*	Southern Taiwan	significantly inhibit the accumulation of the pro-inflammatory iNOS and COX-2 protein of the lipopolysaccharide (LPS)-stimulated RAW264.7 macrophage cells; inactive against the MOLT-4, SUP-T1, U-937, DLD-1, LNCaP, and MCF7 cell lines at 20 μg/mL	[23]
**49**	2*β*-hydroxy-7*β*,8*α*-epoxynephthenol	cembrane diterpene	*N. columnaris*	Southern Taiwan	inactive against the MOLT-4, SUP-T1, U-937, DLD-1, LNCaP, and MCF7 cell lines at 20 μg/mL	[48]
**50**	2*β*-hydroxy-11*α*,12*β*-epoxynephthe	cembrane	*N. columnaris*	Southern Taiwan	inactive against the MOLT-4, SUP-T1, U-937, DLD-1,	[48]

No.	Name	Class	Species	Locality	Bioassays	Ref.
	nol	diterpene			LNCaP, and MCF7 cell lines at 20 μg/mL	
**51**	epoxynephthenol	cembrane diterpene	*N. columnaris*	Southern Taiwan	inactive against the MOLT-4, SUP-T1, U-937, DLD-1, LNCaP, and MCF7 cell lines at 20 μg/mL	[48]
**52**	10-hydroxy-nephthenol acetate	cembrane diterpene	*Nephthea* sp.	Layangan, Sabah, Malaysia	antibacterial activity against *S. aureus* and *E. coli* with a MBC/MIC ratio of 2.4 and 3.0, respectively; cytotoxic activities against the Hela and MCF-7 cell lines (IC_{50} 40.0 and 25.0 μg/mL, respectively)	[24]
**53**	7,8-epoxy-10-hydroxy-nephthenol acetate	cembrane diterpene	*Nephthea* sp.	Layangan, Sabah, Malaysia	antibacterial activity against *S. aureus* and *E. coli* with a MBC/MIC ratio of 6.0 and 6.0, respectively; cytotoxic activities against the Hela and MCF-7 cell lines (IC_{50} 125.0 and 75.0 μg/mL, respectively)	[24]
**54**	6-acetoxy-7,8-epoxy-10-hydroxy-nephthenol acetate	cembrane diterpene	*Nephthea* sp.	Layangan, Sabah, Malaysia	inactive against the bacterial strains *S. aureus* ATCC 6538, *Listeria monocytogenes* ATCC 19113, *E. coli* ATCC 35210, and *Salmonella typhimurium* ATCC 13311, and the HeLa, MCF-7 and HT-29 cell lines	[24]
**55**	6-acetoxy-7,8-epoxy-nephthenol acetate	cembrane diterpene	*Nephthea* sp.	Layangan, Sabah, Malaysia	inactive against the bacterial strains *S. aureus* ATCC 6538, *L. monocytogenes* ATCC 19113, *E. coli* ATCC 35210, and *S. typhimurium* ATCC 13311, and the HeLa, MCF-7 and HT-29 cell lines	[24]
			Nephthea sp.	Sepanggar Bay, Sabah, North Borneo, Malaysia	antifungal activities against *Exophiala* sp. NJM 1551, *H. sabahensis* IPMB 1402, *H. milfordensis* IPMB 1603, and *L. thermophilum* IPMB 1401 (MIC 50, 25, 50, and 25 μg/mL, respectively), inactive against *F. moniliforme* NJM 8995, *F. oxysporum* NJM 0179 and *F. solani* NJM 8996 (all MIC >100 μg/mL)	[25]
**56**	nephthecrassocolide A	cembrane diterpene	*Nephthea* sp.	Sepanggar Bay, Sabah, North Borneo, Malaysia	antifungal activities against *Exophiala* sp. NJM 1551, *F. oxysporum* NJM 0179, *F. solani* NJM 8996, *H. sabahensis* IPMB 1402, *H. milfordensis* IPMB 1603, and *L. thermophilum* IPMB 1401 (MIC 25, 50, 50, 25, 25, and 25 μg/mL, respectively), inactive against *F. moniliforme* NJM 8995	[25]

No.	Name	Class	Species	Locality	Bioassays	Ref.
					(MIC >100 μg/mL)	
**57**	nephthecrassocolide B	cembrane diterpene	*Nephthea* sp.	Sepanggar Bay, Sabah, North Borneo, Malaysia	antifungal activities against *Exophiala* sp. NJM 1551, *H. sabahensis* IPMB 1402, *H. milfordensis* IPMB 1603, and *L. thermophilum* IPMB 1401 (MIC 50, 50, 50, and 25 μg/mL, respectively), inactive against *F. moniliforme* NJM 8995, *F. oxysporum* NJM 0179 and *F. solani* NJM 8996 (all MIC >100 μg/mL)	[25]
**58**	6-acetoxy nephthenol acetate	cembrane diterpene	*Nephthea* sp.	Sepanggar Bay, Sabah, North Borneo, Malaysia	antifungal activities against *Exophiala* sp. NJM 1551, *H. sabahensis* IPMB 1402, *H. milfordensis* IPMB 1603, and *L. thermophilum* IPMB 1401 (MIC 50, 25, 25, and 25 μg/mL, respectively), inactive against *F. moniliforme* NJM 8995, *F. oxysporum* NJM 0179 and *F. solani* NJM 8996 (all MIC >100 μg/mL)	[25]
**59**	epoxy nephthenol acetate	cembrane diterpene	*Nephthea* sp.	Sepanggar Bay, Sabah, North Borneo, Malaysia	antifungal activities against *Exophiala* sp. NJM 1551, *F. moniliforme* NJM 8995, *F. oxysporum* NJM 0179, *F. solani* NJM 8996, *H. sabahensis* IPMB 1402, *H. milfordensis* IPMB 1603, and *L. thermophilum* IPMB 1401 (MIC 25, 50, 50, 50, 25, 25, and 25 μg/mL, respectively)	[25]
**60**	litophynin A	eunicellane diterpene	*Litophyton* sp.	Sukumo Bay, Kochi Prefecture, Japan	insect growth inhibitory against the silkworm *Bombyx mori* L. (ED_{50} 12 ppm)	[49]
**61**	litophynin B	eunicellane diterpene	*Litophyton* sp.	Sukumo Bay, Kochi Prefecture, Japan	insect growth inhibitory against the silkworm *B. mori* L. (ED_{50} 2.7 ppm)	[49]
**62**	litophynin C	eunicellane diterpene	*Litophyton* sp.	Sukumo Bay, Kochi Prefecture, Japan	insect growth inhibitory against the second instar larvae of the silkworm *B. mori* L. (ED_{50} 25 ppm)	[50]
			Nephthea sp.	Sharm El-Sheikh, Egypt (Red Sea)	potential SARS-CoV-2 main protease inhibitor *in silica*	[45]
**63**	litophynin D	eunicellane diterpene	*Litophyton* sp.	Sukumo Bay, Kochi Prefecture, Japan	significant brine shrimp lethalty (LD_{50} 0.9 ppm)	[51]
**64**	litophynin E	eunicellane diterpene	*Litophyton* sp.	Sukumo Bay, Kochi Prefecture, Japan	[a] –	[51]

No.	Name	Class	Species	Locality	Bioassays	Ref.
			Litophyton sp.	Nango-cho, Miyazaki Prefecture, Japan	[b] positive in a hemolytic reaction test	[54]
**65**	litophynin F	eunicellane diterpene	*Litophyton* sp.	Sukumo Bay, Kochi Prefecture, Japan	inactive against the second instar larvae of the silkworm *B. mori* L. at 100 ppm)	[52]
			Litophyton viscudium	Otsuki Town, Kochi Prefecture, Japan	cytotoxic activity against the HL-60 cell line (IC_{50} 18 μM)	[18]
**66**	litophynin G	eunicellane diterpene	*Litophyton* sp.	Sukumo Bay, Kochi Prefecture, Japan	insect growth inhibitory against the second instar larvae of the silkworm *B. mori* L. (ED_{50} 42 ppm)	[52]
**67**	litophynin H	eunicellane diterpene	*Litophyton* sp.	Sukumo Bay, Kochi Prefecture, Japan	inactive against the second instar larvae of the silkworm *B. mori* L. at 100 ppm	[52]
			Litophyton sp.	Nango-cho, Miyazaki Prefecture, Japan	[b] positive in a hemolytic reaction test	[54]
**68**	litophynin I	eunicellane diterpene	*Litophyton* sp.	Sukumo Bay, Kochi Prefecture, Japan	significant molluscicidal activity against the muricid gastropod *Drupella fragum* with 100% mortality within 24 hours at 30 ppm, repellent activity against the gastropod when impregnated on filterpaper by 45 μg/cm^2.	[53]
**69**	litophynin J	eunicellane diterpene	*Litophyton* sp.	Sukumo Bay, Kochi Prefecture, Japan	significant molluscicidal activity against the muricid gastropod *D. fragum* with 100% mortality within 24 hours at 30 ppm, repellent activity against the gastropod when impregnated on filterpaper by 45 μg/cm^2.	[53]
**70**	litophynol A	eunicellane diterpene	*Litophyton* sp.	Nango-cho, Miyazaki Prefecture, Japan	[b] positive in a hemolytic reaction test	[54]
**71**	litophynol B	eunicellane diterpene	*Litophyton* sp.	Nango-cho, Miyazaki Prefecture, Japan	[b] positive in a hemolytic reaction test	[54]
**72**	litophynin I monoacetate	eunicellane diterpene	*Litophyton* sp.	Nango-cho, Miyazaki Prefecture, Japan	[b] positive in a hemolytic reaction test	[54]
**73**	[c] 6-oxo litophynin H	eunicellane diterpene	*L. viscudium*	Otsuki Town, Kochi Prefecture, Japan	cytotoxic activity against the HL-60 cell line (IC_{50} 20 μM)	[18]
**74**	[c] 6-oxo litophynin H 12-acetate	eunicellane diterpene	*L. viscudium*	Otsuki Town, Kochi Prefecture, Japan	cytotoxic activity against the HL-60 cell line (IC_{50} 20 μM)	[18]

No.	Name	Class	Species	Locality	Bioassays	Ref.
**75**	[c] 6-oxo litophynol A	eunicellane diterpene	*L. viscudium*	Otsuki Town, Kochi Prefecture, Japan	cytotoxic activity against the HL-60 cell line (IC_{50} 5.7 μM)	[18]
**76**	[c] 6-*epi* litophynol A	eunicellane diterpene	*L. viscudium*	Otsuki Town, Kochi Prefecture, Japan	cytotoxic activity against the HL-60 cell line (IC_{50} 4.2 μM)	[18]
**77**	[c] 6-methyl litophynin E	eunicellane diterpene	*L. viscudium*	Otsuki Town, Kochi Prefecture, Japan	cytotoxic activity against the HL-60 cell line (IC_{50} 50 μM)	[18]
**78**	lemnabourside	serrulatane diterpene	*L. setoensis*	Singaraja, Bali Island, Indonesia	weak cytotoxic activities against the HT-29, Capan-1, A549, and SNU-398 cell lines (all IC_{50} >20 μM)	[17]
**79**	biflora-4,9,15-triene	serrulatane diterpene	*L. setoensis*	Singaraja, Bali Island, Indonesia	weak cytotoxic activities against the HT-29, Capan-1, A549, and SNU-398 cell lines (all IC_{50} >20 μM)	[17]
**80**	litosetoenin A	5,9-cyclized serrulatane diterpene	*L. setoensis*	Singaraja, Bali Island, Indonesia	weak cytotoxic activities against the HT-29, Capan-1, A549, and SNU-398 cell lines (all IC_{50} >20 μM)	[17]
**81**	litosetoenin B	5,9-cyclized serrulatane diterpene	*L. setoensis*	Singaraja, Bali Island, Indonesia	weak cytotoxic activities against the HT-29, Capan-1, A549, and SNU-398 cell lines (all IC_{50} >20 μM)	[17]
**82**	litosetoenin C	5,9-cyclized serrulatane diterpene	*L. setoensis*	Singaraja, Bali Island, Indonesia	weak cytotoxic activities against the HT-29, Capan-1, A549, and SNU-398 cell lines (all IC_{50} >20 μM)	[17]
**83**	litosetoenin D	5,9-cyclized serrulatane diterpene	*L. setoensis*	Singaraja, Bali Island, Indonesia	weak cytotoxic activities against the HT-29, Capan-1, A549, and SNU-398 cell lines (all IC_{50} >20 μM)	[17]
**84**	litosetoenin E	5,9-cyclized serrulatane diterpene	*L. setoensis*	Singaraja, Bali Island, Indonesia	weak cytotoxic activities against the HT-29, Capan-1, A549, and SNU-398 cell lines (all IC_{50} >20 μM)	[17]
**85**	chabrolin A	chabrolane diterpene	*Nephthea chablrolii*	Green Islang, Taitong County, Taiwan	cytotoxicity against the P-388 cell line (ED_{50} 3.18 μg/mL), inactive aginst the A549 and HT-29 cell lines and HCMV	[56]
**86**	pacificin H	prenylbicyclogermacrane diterpene	*Nephthea* sp.	Sharm El-Sheikh, Egypt (Red Sea)	potential SARS-CoV-2 main protease inhibitor *in silica*	[45]
**87**	chabrolene	norditerpene	*Nephthea* sp.	Mantanani Island,	repellent activity against the maize weevil *S. zeamais* at 25	[46]

No.	Name	Class	Species	Locality	Bioassays	Ref.
				Sabah, Malaysia	μg/cm^2	
**88**	all-*trans*-peridinin	tetraterpene	*L. arboreum*	Hurghada, Egypt (Red Sea)	antiproliferative activities against the HUVEC and K-562cell lines (GI_{50} 48.4±1.2 and 53.8±3.9 μM, respectively), cytotoxicity against the HeLa cell line (IC_{50} 51.9±0.7 μM)	[47]
**89**	*O*-methylisogrifolin	meroterpene	*Nephthea* sp.	Sharm El-Sheikh, Egypt (Red Sea)	potential SARS-CoV-2 main protease inhibitor *in silica*	[45]
**90**	chabrolobenzoquinone E	meroterpene	*Nephthea* sp.	Sharm El-Sheikh, Egypt (Red Sea)	potential SARS-CoV-2 main protease inhibitor *in silica*	[45]
**91**	chabrolohydroxybenzoquinone E	meroterpene	*Nephthea* sp.	Sharm El-Sheikh, Egypt (Red Sea)	potential SARS-CoV-2 main protease inhibitor *in silica*	[45]
**92**	chabrolonaphthoquinone A	meroterpene	*Nephthea* sp.	Sharm El-Sheikh, Egypt (Red Sea)	potential SARS-CoV-2 main protease inhibitor *in silica*	[45]
**93**	nephthoside monoacetate	meroterpene	*Nephthea* sp.	Sharm El-Sheikh, Egypt (Red Sea)	potential SARS-CoV-2 main protease inhibitor *in silica*	[45]
**94**	4*α*-methyl-3*β*,8*β*-dihydroxy-5*α*-ergost-24(28)-en-23-one	4*α*-methylated steroid	*L. viridis*	Lesser Sunda Islands, Indonesia	[a] –	[58]
			Nephthea sp.	Hurghada, Egypt (Red Sea)	cytotoxicity against the MCF-7 cell line (IC_{50} 124.3 μg/mL)	[42]
			Nephthea sp.	Mantanani Island, Sabah, Malaysia	no repellent activity against the maize weevil *S. zeamais* at 250 μg/cm^2	[46]
**95**	4*α*,24-dimethyl-5*α*-cholest-24(28)-en-3*β*,8*β*,18-triol	4*α*-methylated steroid	*Litophyton mollis*	Hurghada, Egypt (Red Sea)	cytotoxic activities against the K562 and A549 cell lines (IC_{50} 8.9±0.9 and 25.7±1.5 μM, respectively)	[59]
**96**	(22*E*,24*R*)-4*α*,24-dimethyl-5*α*-cholest-22-en-3*β*,8*β*,18-triol	4*α*-methylated steroid	*L. mollis*	Hurghada, Egypt (Red Sea)	inactive against the K562 and A549 cell lines (both IC_{50} >50 μM)	[59]
			Nephthea sp.	Hurghada, Egypt (Red Sea)	cytotoxicity against the MCF-7 cell line (IC_{50} 113.6 μg/mL)	[42]
**97**	(22*E*)-4*α*,24-dimethyl-5*α*-cholesta-22,24(28)-dien-3*β*,8*β*,18-triol	4*α*-methylated steroid	*L. mollis*	Hurghada, Egypt (Red Sea)	inactive against the K562 and A549 cell lines (both IC_{50} >50 μM)	[59]
**98**	nebrosteroid M	4*α*-methylated	*L. mollis*	Hurghada, Egypt	cytotoxic activity against the A549 cell line (IC_{50} 20.4±1.1	[59]

No.	Name	Class	Species	Locality	Bioassays	Ref.
		steroid		(Red Sea)	μM), inactive against the K562 cell line (IC_{50} >50 μM)	
			L. arboreum	Hurghada, Egypt (Red Sea)	cytotoxic activity against the A549 and MCF-7 cell lines (IC_{50} 36.9±2.9 and 55.3±4.9 μmol/mL, respectively), inactive against the HepG2 cell line (IC_{50} >100 μmol/mL)	[41]
			L. arboreum	Neweba, Egypt (Red Sea)	no anti-malarial activity against chloroquine-sensitive *P. falciparum* D6 and chloroquine-resistant *P. falciparum* W2 at the concentration of 4.76 mg/mL, non-toxic to the Vero cell line at the concentration of 4.76 mg/mL	[19]
**99**	(22*E*,24*R*)-4*α*,24-dimethyl-5*α*-cholest-22-en-3*β*,8*β*,11*β*-triol	4*α*-methylated steroid	*L. mollis*	Hurghada, Egypt (Red Sea)	cytotoxic activities against the K562 and A549 cell lines (IC_{50} 7.7±0.8 and 20.8±1.2 μM, respectively)	[59]
			L. arboreum	Neweba, Egypt (Red Sea)	no anti-malarial activity against chloroquine-sensitive *P. falciparum* D6 and chloroquine-resistant *P. falciparum* W2 at the concentration of 4.76 mg/mL, non-toxic to the Vero cell line at the concentration of 4.76 mg/mL	[19]
**100**	nebrosteroid D	4*α*-methylated steroid	*L. mollis*	Hurghada, Egypt (Red Sea)	cytotoxic activities against the K562 and A549 cell lines (IC_{50} 6.0±0.5 and 22.1±1.4 μM, respectively)	[59]
**101**	nebrosteroid A	4*α*-methylated steroid	*L. mollis*	Hurghada, Egypt (Red Sea)	cytotoxic activity against the K562 cell line (IC_{50} 5.8±0.8 μM)	[59]
**102**	23*ξ*-acetoxy-4*α*,24-dimethyl-5*α*-cholest-24(28)-en-3*β*,8*β*,11*β*-triol	4*α*-methylated steroid	*L. mollis*	Hurghada, Egypt (Red Sea)	cytotoxic activity against the K562 cell line (IC_{50} 5.6±1.2 μM)	[59]
**103**	(22*E*,24*R*)-4*α*,23,24-trimethyl-5*α*-cholest-22-en-3*β*,8*β*,11*β*-triol	4*α*-methylated steroid	*L. mollis*	Hurghada, Egypt (Red Sea)	inactive against the K562 and A549 cell lines (both IC_{50} >50 μM)	[59]
			L. arboreum	Neweba, Egypt (Red Sea)	no anti-malarial activity against chloroquine-sensitive *P. falciparum* D6 and chloroquine-resistant *P. falciparum* W2 at the concentration of 4.76 mg/mL, non-toxic to the Vero cell line at the concentration of 4.76 mg/mL	[19]
**104**	(22*Z*)-4*α*,24*ξ*-dimethyl-5*α*-cholest-22-en-3*β*,8*β*,11*β*-triol	4*α*-methylated steroid	*L. mollis*	Hurghada, Egypt (Red Sea)	[a] –	[59]
**105**	4*α*,24*R*-dimethyl-5*α*-cholest-22-en-3*β*-ol	4*α*-methylated steroid	*Nephthea* sp.	Hurghada, Egypt (Red Sea)	cytotoxicity against the MCF-7 cell line (IC_{50} 201.7 μg/mL)	[42]

No.	Name	Class	Species	Locality	Bioassays	Ref.
**106**	4α,24-dimethyl-cholest-22*Z*-en-3β-ol	4α-methylated steroid	*L. arboreum*	Hurghada, Egypt (Red Sea)	[a]–	[64]
**107**	4α-methylergosta-22(*E*),24(28)-dien-3β-ol	4α-methylated steroid	*N. columnaris*	Southern Taiwan	[a]–	[65]
**108**	4α-methyl-ergosta-6,8(14),22*E*-triene-3β-ol	4α-methylated steroid	*Nephthea* sp.	Sharm El-Sheikh, Egypt (Red Sea)	potential SARS-CoV-2 main protease inhibitor *in silica*	[45]
**109**	24-methylenecholest-5-en-3β,7β,19-triol	ergostane-type and related steroid	*L. viridis*	Serwaru, Leti Island, Indonesia	[a]–	[57]
			L. arboreum	Xisha Islands, Hainan, China (South China Sea)	[a]–	[68]
			L. arboreum	Sharm El-Sheikh, Egypt (Red Sea)	cytotoxic activities against the HeLa, Vero and U937 cell lines (IC_{50} 8±0.5, 11.4±0.04 and 16.4±1.25 μM, respectively)	[38]
			L. arboreum	Hurghada, Egypt (Red Sea)	[a]–	[64]
			L. arboreum	Sharm El-Sheikh, Egypt (Red Sea)	cytotoxic effects against the HepG2, MDA and A549 cancer cell lines (IC_{50} 8.5, 5.5 and 9.3 μg/mL, respectively)	[39]
			L. arboreum	Jeddah, Saudi Arabia (Red Sea)	[a]–	[20]
			Nephthea sp.	Hurghada, Egypt (Red Sea)	cytotoxicity against the MCF-7 cell line (IC_{50} 56.6 μg/mL)	[42]
**110**	7-acetoxy-24-methylenecholest-5-en-3β,7β,19-triol	ergostane-type and related steroid	*L. viridis*	Serwaru, Leti Island, Indonesia	[a]–	[57]
			L. arboreum	Sharm El-Sheikh, Egypt (Red Sea)	inhibitory activity against HIV-1 protease receptor (IC_{50} 4.85±0.18 μM), cytotoxic activities against the HeLa, Vero and U937 cell lines (IC_{50} 5.3±0.60, 31.3±14.03 and 10.6±0.12 μM, respectively)	[38]
			L. arboreum	Sharm El-Sheikh, Egypt (Red Sea)	cytotoxic effects against the HepG2, MDA and A549 cancer cell lines (IC_{50} 6.07, 6.3 and 6.2 μg/mL, respectively)	[39]
			L. arboreum	Neweba, Egypt	[a]–	[19]

No.	Name	Class	Species	Locality	Bioassays	Ref.
				(Red Sea)		
			L. arboreum	Jeddah, Saudi Arabia (Red Sea)	[b] antiproliferative effects toward MCF-7, HCT116 and HepG2 cell lines	[43]
			Nephthea sp.	Hurghada, Egypt (Red Sea)	cytotoxicity against the MCF-7 cell line (IC_{50} 37.0 μg/mL)	[42]
**111**	litosterol	ergostane-type and related steroid	*L. viridis*	Ishiqaki Isoland, Okinawa, Japan	[a] –	[67]
			L. arboreum	Hurghada, Egypt (Red Sea)	[a] –	[64]
			L. arboreum	Neweba, Egypt (Red Sea)	[a] –	[19]
			N. columnaris	Southern Taiwan	no inhibitory effects on the generation of superoxide anions and the release of elastase (IC_{50} >10 μM)	[74]
**112**	5,6-epoxylitosterol	ergostane-type and related steroid	*L. viridis*	Ishiqaki Isoland, Okinawa, Japan	antileukemic activity against the P388 cell line (IC_{50} 0.5 μg/mL)	[67]
			N. columnaris	Southern Taiwan	inhibitory effects on the generation of superoxide anions and the release of elastase (IC_{50} 4.60±0.85 and 3.90±0.88 μM, respectively)	[74]
**113**	(24*E*)-24-ethyl-5*α*-cholesta-8,24(28)-diene-3*β*,12*β*,19-triol	ergostane-type and related steroid	*L. arboreum*	Xisha Islands, Hainan, China (South China Sea)	[a] –	[68]
**114**	24-methylcholesta-5,24(28)-diene-3*β*-ol	ergostane-type and related steroid	*L. arboreum*	Sharm El-Sheikh, Egypt (Red Sea)	cytotoxic activities against the HeLa and Vero cell lines (IC_{50} 48±8.7 and 100±1.2 μM, respectively), inactive against the U937 cell line (IC_{50} >100 μM)	[38]
			L. arboreum	Hurghada, Egypt (Red Sea)	[a] –	[64]
			L. arboreum	Jeddah, Saudi Arabia (Red Sea)	[a] –	[20]
			L. arboreum	Hurghada, Egypt (Red Sea)	cytotoxic activities against the A549, MCF-7 and HepG2 cell lines (IC_{50} 28.5±4.4, 70.0±2.3 and 77.6±3.3 μmol/mL,	[41]

No.	Name	Class	Species	Locality	Bioassays	Ref.
					respectively)	
			Nephthea sp.	Hurghada, Egypt (Red Sea)	cytotoxicity against the MCF-7 cell line (IC_{50} 339.2 μg/mL)	[42]
			N. erecta	Pingtung, Taiwan	inactive against the K-562, MOLT-4, SUP-T1, and U-937 cell lines (all IC_{50} >20 μM)	[75]
**115**	3*β*,7*β*-dihydroxy-24-methylenecholesterol	ergostane-type and related steroid	*L. arboreum*	Neweba, Egypt (Red Sea)	anti-malarial activity against chloroquine-resistant *P. falciparum* W2 (IC_{50} 4.0 mg/mL), no anti-malarial effect againste hloroquine-sensitive *P. falciparum* D6 at the concentration of 4.76 mg/mL	[19]
**116**	chabrolosteroid I	ergostane-type and related steroid	*L. arboreum*	Neweba, Egypt (Red Sea)	no anti-malarial effect against chloroquine-resistant *P. falciparum* W2 and chloroquine-sensitive *P. falciparum* D6 at the concentration of 4.76 mg/mL	[19]
**117**	13,14-*seco*-22-norergosta-4,24(28)-dien-19-hydroperoxide-3-one	ergostane-type and related steroid	*L. arboreum*	Jeddah, Saudi Arabia (Red Sea)	[b] antiproliferative effects toward MCF-7, HCT116 and HepG2 cell lines	[43]
**118**	sarcsteroid F	ergostane-type and related steroid	*Litophyton* sp.	Egyptian Red Sea	[a] –	[27]
**119**	24-methylenecholestane-1*α*,3*β*,5*α*,6*β*,11*α*-pentol-11-monoacetate	ergostane-type and related steroid	*Litophyton* sp.	Egyptian Red Sea	[a] –	[27]
**120**	nebrosteroid O	ergostane-type and related steroid	*N. chabrolii*	San-Hsian-Tai, Taitong County, Taiwan	cytotoxic activities against the A549, HT-29 and P-388 cell lines (ED_{50} 5.9, 5.9 and 1.2 μg/mL, respectively); inactive against HCMV (ED_{50} >100 μg/mL)	[69]
			Nephthea sp.	Sharm El-Sheikh, Egypt (Red Sea)	potential SARS-CoV-2 main protease inhibitor *in silica*	[45]
**121**	nebrosteroid P	ergostane-type and related steroid	*N. chabrolii*	San-Hsian-Tai, Taitong County, Taiwan	cytotoxic activities against the A549, HT-29 and P-388 cell lines (ED_{50} 7.2, 9.5 and 1.7 μg/mL, respectively); inactive against HCMV (ED_{50} >100 μg/mL)	[69]
**122**	nebrosteroid Q	ergostane-type and related steroid	*N. chabrolii*	San-Hsian-Tai, Taitong County, Taiwan	cytotoxic activities against the A549, HT-29 and P-388 cell lines (ED_{50} 6.1, 8.0 and 1.1 μg/mL, respectively); inactive against HCMV (ED_{50} >100 μg/mL)	[70]
**123**	nebrosteroid R	ergostane-type	*N. chabrolii*	San-Hsian-Tai, Taitong	cytotoxic activities against the A549, HT-29 and P-388 cell	[70]

No.	Name	Class	Species	Locality	Bioassays	Ref.
		and related steroid		County, Taiwan	lines (ED_{50} 11.4, 20.9 and 1.2 μg/mL, respectively); inactive against HCMV (ED_{50} >100 μg/mL)	
**124**	nebrosteroid S	ergostane-type and related steroid	*N. chabrolii*	San-Hsian-Tai, Taitong County, Taiwan	cytotoxic activities against the A549, HT-29 and P-388 cell lines (ED_{50} 8.7, 15.3 and 1.0 μg/mL, respectively); inactive against HCMV (ED_{50} >100 μg/mL)	[70]
**125**	nephalsterol A	ergostane-type and related steroid	*N. columnaris*	Southern Taiwan	cytotoxicities against the MOLT-4, SUP-T1, U-937, DLD-1, LNCaP, and MCF7 cell lines (IC_{50} 22.5, 32.4, 38.6, 44.2, 11.6, and 9.8 μM, respectively)	[48]
**126**	columnaristerol A	ergostane-type and related steroid	*N. columnaris*	Southern Taiwan	moderate cytotoxic effects against the MOLT-4 and SUP-T1 cell lines (IC_{50} 18.3 and 25.5 μM, respectively), inactive against the K-562 and U-937 cell lines at 20 μg/mL	[73]
**127**	columnaristerol B	ergostane-type and related steroid	*N. columnaris*	Southern Taiwan	no inhibitory effects on the generation of superoxide anions and the release of elastase (IC_{50} >10 μM)	[74]
**128**	columnaristerol C	ergostane-type and related steroid	*N. columnaris*	Southern Taiwan	no inhibitory effects on the generation of superoxide anions and the release of elastase (IC_{50} >10 μM)	[74]
**129**	nephtheasteroid A	ergostane-type and related steroid	*N. erecta*	Pingtung, Taiwan	inactive against the K-562, MOLT-4, SUP-T1, and U-937 cell lines (all IC_{50} >20 μM)	[75]
**130**	nephtheasteroid B	ergostane-type and related steroid	*N. erecta*	Pingtung, Taiwan	inactive against the K-562, MOLT-4, SUP-T1, and U-937 cell lines (IC_{50} >20 μM)	[75]
**131**	erectasteroid F	ergostane-type and related steroid	*N. erecta*	Pingtung, Taiwan	cytotoxic activities against the K-562, MOLT-4, SUP-T1, and U-937 cell lines (IC_{50} 6.5, 8.0, 8.0, and 12.9 μM, respectively)	[75]
**132**	(3*β*,7*β*)-ergost-5,24(28)-diene-3*β*,7*β*, 19-triol-7,19-diacetate	ergostane-type and related steroid	*N. erecta*	Pingtung, Taiwan	cytotoxic activities against the K-562, MOLT-4, SUP-T1, and U-937 cell lines (IC_{50} 14.0, 7.9, 7.3, and 6.8 μM, respectively)	[75]
**133**	24-methyl-cholesta-5,24(28)-diene-3*β*,19-diol-7*β*-monoacetate	ergostane-type and related steroid	*N. erecta*	Pingtung, Taiwan	cytotoxic activities against the K-562, MOLT-4 and SUP-T1 cell lines (IC_{50} 11.2, 19..9 and 16.3 μM), inactive against the U-937 cell line (IC_{50} >20 μM)	[75]
			N. erecta	Pingtung, Taiwan	inhibited human small cell lung cancer growth via apoptosis induction	[76]

No.	Name	Class	Species	Locality	Bioassays	Ref.
**134**	armatinol B	ergostane-type and related steroid	*N. erecta*	Pingtung, Taiwan	inactive against the K-562, MOLT-4, SUP-T1, and U-937 cell lines (all IC_{50} >20 μM)	[75]
**135**	chabrolosteroid C	ergostane-type and related steroid	*Nephthea* sp.	Sharm El-Sheikh, Egypt (Red Sea)	potential SARS-CoV-2 main protease inhibitor *in silica*	[45]
**136**	ergost-5,25-diene-3*β*,24*S*,28-triol	ergostane-type and related steroid	*Nephthea* sp.	Sharm El-Sheikh, Egypt (Red Sea)	potential SARS-CoV-2 main protease inhibitor *in silica*	[45]
**137**	nebrosteroid N	cholestane-type and related steroid	*Nephthea chabrolii*	San-Hsian-Tai, Taitong County, Taiwan	cytotoxic activities against the A549, HT-29 and P-388 cell lines (ED_{50} 6.7, 9.5 and 0.9 μg/mL, respectively); inactive against HCMV (ED_{50} >100 μg/mL)	[69]
**138**	(20*S*,22*R*)-progesterone-1,4-diene-22-acetyl-3-one	cholestane-type and related steroid	*Nephthea* sp.	Naozhou Island, South China Sea	[b] weak cytotoxic activities against A549 and Hepg2 cell lines	[77]
**139**	(12*β*,22*R*)-12-acetoxy-22-hydroxy-cholesta-1,4-dien-3-one	cholestane-type and related steroid	*Nephthea* sp.	Naozhou Island, South China Sea	cytotoxic activity against the HeLa cell line (IC_{50} 7.51±0.22 μg/mL)	[78]
**140**	(12*β*,22*R*)-12-hydroxy-22-acetoxy-cholesta-1,4-dien-3-one	cholestane-type and related steroid	*Nephthea* sp.	Naozhou Island, South China Sea	cytotoxic activity against the HeLa cell line (IC_{50} 7.50±0.31 μg/mL)	[78]
**141**	(12*β*,22*R*)-12,22-diacetoxy-cholesta-1,4-dien-3-one	cholestane-type and related steroid	*Nephthea* sp.	Naozhou Island, South China Sea	cytotoxic activity against the HeLa cell line (IC_{50} 18.48±0.56 μg/mL)	[78]
**142**	(22*R*)-18,22-diacetoxy-cholesta-1,4-dien-3-one	cholestane-type and related steroid	*Nephthea* sp.	Naozhou Island, South China Sea	cytotoxic activity against the HeLa cell line (IC_{50} 8.29±0.42 μg/mL)	[78]
**143**	(20*R*,22*R*)-20-hydroxy-22-acetoxy-cholesta-1,4-dien-3-one	cholestane-type and related steroid	*Nephthea* sp.	Naozhou Island, South China Sea	cytotoxic activity against the HeLa cell line (IC_{50} 17.25±0.61 μg/mL)	[78]
**144**	astrogorgol N	cholestane-type and related steroid	*Nephthea* sp.	Naozhou Island, South China Sea	cytotoxic activity against the HeLa cell line (IC_{50} 18.72±0.78 μg/mL)	[78]
**145**	nephthoacetal	cholestane-type and related steroid	*Nephthea* sp.	Naozhou Island, South China Sea	strong antifouling activity against the settlement of *Bugula neritina* larvae (EC_{50} 2.5 μg/mL), moderate cytotoxicity against the HeLa cell line (EC_{50} 12.3 μg/mL)	[79]
**146**	dendronesterone C	cholestane-type and related steroid	*Nephthea* sp.	Sharm El-Sheikh, Egypt (Red Sea)	potential SARS-CoV-2 main protease inhibitor *in silica*	[45]
**147**	(17*α*)-pregnan-4-ene-3,20-dione	pregnane-type and related steroid	*Nephthea* sp.	Naozhou Island, South China Sea	[a] –	[80]

No.	Name	Class	Species	Locality	Bioassays	Ref.
**148**	(20*S*)-pregnan-1,4-diene-3-oxo-20-carboxylic acid methyl ester	pregnane-type and related steroid	*Nephthea* sp.	Naozhou Island, South China Sea	[a] –	[80]
**149**	pregnan-4-ene-3,6,20-trione	pregnane-type and related steroid	*Nephthea* sp.	Naozhou Island, South China Sea	[a] –	[80]
**150**	(20*R*)-pregnan-4,16-dien-20-hydroxy-3-one	pregnane-type and related steroid	*Nephthea* sp.	Naozhou Island, South China Sea	[a] –	[80]
**151**	pregnan-15*β*-hydroxy-4,6-diene-3,20-dione	pregnane-type and related steroid	*Nephthea* sp.	Naozhou Island, South China Sea	[a] –	[80]
**152**	pregnan-4,6-diene-3,20-dione	pregnane-type and related steroid	*Nephthea* sp.	Naozhou Island, South China Sea	[a] –	[80]
**153**	erythro-*N*-dodecanoyl-docosasphinga-(4*E*,8*E*)-dienine	ceramide	*L. arboreum*	Sharm El-Sheikh, Egypt (Red Sea)	cytotoxic activity against the HeLa cell line (IC_{50} 38.17±0.7 μM), inactive against the Vero and U937 cell lines (both IC_{50} >100 μM)	[38]
			L. arboreum	Jeddah, Saudi Arabia (Red Sea)	[b] antiproliferative effects toward MCF-7, HCT116 and HepG2 cell lines	[43]
			L. arboreum	Jeddah, Saudi Arabia (Red Sea)	[a] –	[40]
			L. arboreum	Neweba, Egypt (Red Sea)	[a] –	[19]
			Nephthea sp.	Hurghada, Egypt (Red Sea)	cytotoxicity against the MCF-7 cell line (IC_{50} 238.5 μg/mL)	[42]
**154**	erythro-*N*-palmityl-octadecasphinga-4(*E*),8(*E*)-dienine	ceramide	*L. arboreum*	Hurghada, Egypt (Red Sea)	[a] –	[64]
			Nephthea sp.	Sharm El-Sheikh, Egypt (Red Sea)	potential SARS-CoV-2 main protease inhibitor *in silica*	[45]
**155**	thymine	pyrimidine	*L. arboreum*	Jeddah, Saudi Arabia (Red Sea)	[a] –	[40]
			L. arboreum	Hurghada, Egypt (Red Sea)	inactive against the A549, MCF-7 and HepG2 cell lines (all IC_{50} >100 μg/mL), inactive against against *Leishmania major* (IC_{50} >100 μg/mL)	[81]

No.	Name	Class	Species	Locality	Bioassays	Ref.
**156**	thymidine	pyrimidine	*L. arboreum*	Jeddah, Saudi Arabia (Red Sea)	[a] –	[40]
**157**	uracil	pyrimidine	*L. arboreum*	Hurghada, Egypt (Red Sea)	inactive against the A549, MCF-7 and HepG2 cell lines (all IC_{50} >100 μg/mL), inactive against against *L. major* (IC_{50} >100 μg/mL)	[81]
**158**	uridine	pyrimidine	*L. arboreum*	Hurghada, Egypt (Red Sea)	inactive against the A549, MCF-7 and HepG2 cell lines (all IC_{50} >100 μg/mL), inactive against against *L. major* (IC_{50} >100 μg/mL)	[81]
**159**	1,3-dimethly uracil	pyrimidine	*Nephthea* sp.	Naozhou Island, South China Sea	[a] –	[82]
**160**	caffeine	pyrimidine	*Nephthea* sp.	Naozhou Island, South China Sea	[a] –	[82]
**161**	theophylline	pyrimidine	*Nephthea* sp.	Naozhou Island, South China Sea	[a] –	[82]
**162**	cyclo-(Pro-Gly)	peptide	*Nephthea* sp.	Naozhou Island, South China Sea	[a] –	[82]
**163**	leucyl-*N*-methyl-leucyl-leucyl-*N*-methyl-leucine	peptide	*Nephthea* sp.	Naozhou Island, South China Sea	[b] weak cytotoxic activities against A549 and Hepg2 cell lines	[77]
**164**	PGB_2 methyl ester	prostaglandin	*L. arboreum*	gulf of Aqaba, Eilat, Israel (Red Sea)	[a] –	[83]
**165**	litophytolide A	*γ*-lactone	*Litophyton* sp.	Sukumo Bay, Kochi Prefecture, Japan	[a] –	[84]
**166**	litophytolide B	*γ*-lactone	*Litophyton* sp.	Sukumo Bay, Kochi Prefecture, Japan	[a] –	[84]
**167**	[c] (*R*)-5-((*S*,2*Z*,4*E*)-1-hydroxydeca-2,4-dien-1-yl)dihydrofuran-2(3*H*)-one	*γ*-lactone	*L. arboreum*	gulf of Aqaba, Eilat, Israel (Red Sea)	toxic to brine shrimp *Artemia salina* (CC_{50} 15.3 μg/mL), antibacterial activeities against *S. aureus* (diameters of inhibition zone 7.8 mm) and *B. subtilis* (diameters of inhibition zone 5.6 mm), inactive against *E. coli* and *Saccharomyces cerevisiae*	[83]
**168**	[c]	*γ*-lactone	*L. arboreum*	gulf of Aqaba, Eilat,	toxic to brine shrimp *A. salina* (CC_{50} 21.4 μg/mL),	[83]

No.	Name	Class	Species	Locality	Bioassays	Ref.
	(*R*)-5-((*S*,4*E*,8*E*)-1-hydroxy-4,8-dimethyltrideca-4,8-dien-1-yl)-5-methyldihydrofuran-2(3*H*)-one			Israel (Red Sea)	antibacterial activeities against *S. aureus* (diameters of inhibition zone 18.6 mm) and *B. subtilis* (diameters of inhibition zone 14.7 mm), inactive against *E. coli* and *S. cerevisiae*	
**169**	methyl (5Z,8Z,11Z,14Z,17Z)-5,8,11,14,17-icosapentaenoate	fatty acid	*Litophyton* sp.	Sukumo Bay, Kochi Prefecture, Japan	[a] –	[84]
**170**	arachidonic acid	fatty acid	*L. arboreum*	gulf of Aqaba, Eilat, Israel (Red Sea)	[a] –	[83]
**171**	eicosapentaenoic acid	fatty acid	*L. arboreum*	gulf of Aqaba, Eilat, Israel (Red Sea)	[a] –	[83]
**172**	docosahexaenoic acid	fatty acid	*L. arboreum*	gulf of Aqaba, Eilat, Israel (Red Sea)	[a] –	[83]
**173**	chimyl alcohol	glycerol ether	*L. arboreum*	Sharm El-Sheikh, Egypt (Red Sea)	cytotoxic activity against the HeLa and Vero cell lines (IC_{50} 23.35±5.8 and 60±1.14 μM, respectively), inactive against the U937 cell line (both IC_{50} >100 μM), inhibitory activity against HIV-1 protease receptor (IC_{50} 26.6±2.6 μM)	[38]
			L. arboreum	Hurghada, Egypt (Red Sea)	inactive against the A549, MCF-7 and HepG2 cell lines (all IC_{50} >100 μmol/mL)	[41]
**174**	batyl alcohol	glycerol ether	*L. arboreum*	Hurghada, Egypt (Red Sea)	inactive against the A549, MCF-7 and HepG2 cell lines (all IC_{50} >100 μmol/mL)	[41]
**175**	diphenyl selenide	selenide	*Nephthea* sp.	Naozhou Island, South China Sea	[a] –	[82]

[a] The sign '–' indicated no bioassay was recorded in the work.
[b] No specific data was provided in the paper.
[c] Compound was named based on its structure.
